# Advances in hydrogel-mediated gene therapy in ophthalmology: future directions and therapeutic potential

**DOI:** 10.1093/rb/rbag041

**Published:** 2026-03-05

**Authors:** Qinghe Zhang, Ke Yan, Yufei Lv, Tao Tao, Shinan Wu, Zixuan Yang, Linyan Ma, Caihong Huang, Qiuping Liu, Yi Han, Zuguo Liu

**Affiliations:** Xiamen University Affiliated Xiamen Eye Center; Fujian Provincial Key Laboratory of Ophthalmology and Visual Science; Fujian Engineering and Research Center of Eye Regenerative Medicine, Eye Institute of Xiamen University; School of Medicine, Xiamen University, Xiamen, Fujian 361005, China; Department of Ophthalmology, The First Affiliated Hospital of University of South China, Hengyang Medical School, University of South China, Hengyang, Hunan 421001, China; Department of Human Anatomy, Hengyang Medical School, University of South China, Hengyang, Hunan 421001, China; Xiamen University Affiliated Xiamen Eye Center; Fujian Provincial Key Laboratory of Ophthalmology and Visual Science; Fujian Engineering and Research Center of Eye Regenerative Medicine, Eye Institute of Xiamen University; School of Medicine, Xiamen University, Xiamen, Fujian 361005, China; Xiamen University Affiliated Xiamen Eye Center; Fujian Provincial Key Laboratory of Ophthalmology and Visual Science; Fujian Engineering and Research Center of Eye Regenerative Medicine, Eye Institute of Xiamen University; School of Medicine, Xiamen University, Xiamen, Fujian 361005, China; Xiamen University Affiliated Xiamen Eye Center; Fujian Provincial Key Laboratory of Ophthalmology and Visual Science; Fujian Engineering and Research Center of Eye Regenerative Medicine, Eye Institute of Xiamen University; School of Medicine, Xiamen University, Xiamen, Fujian 361005, China; Department of Ophthalmology, The First Affiliated Hospital of University of South China, Hengyang Medical School, University of South China, Hengyang, Hunan 421001, China; Xiamen University Affiliated Xiamen Eye Center; Fujian Provincial Key Laboratory of Ophthalmology and Visual Science; Fujian Engineering and Research Center of Eye Regenerative Medicine, Eye Institute of Xiamen University; School of Medicine, Xiamen University, Xiamen, Fujian 361005, China; Department of Ophthalmology, The First Affiliated Hospital of University of South China, Hengyang Medical School, University of South China, Hengyang, Hunan 421001, China; Department of Ophthalmology, The First Affiliated Hospital of University of South China, Hengyang Medical School, University of South China, Hengyang, Hunan 421001, China; Xiamen University Affiliated Xiamen Eye Center; Fujian Provincial Key Laboratory of Ophthalmology and Visual Science; Fujian Engineering and Research Center of Eye Regenerative Medicine, Eye Institute of Xiamen University; School of Medicine, Xiamen University, Xiamen, Fujian 361005, China; Department of Ophthalmology, The First Affiliated Hospital of University of South China, Hengyang Medical School, University of South China, Hengyang, Hunan 421001, China

**Keywords:** hydrogel, gene therapy, ophthalmology, CRISPR/Cas9, RNAi

## Abstract

Hydrogel-mediated  gene  therapy  has  achieved significant progress in the field of ophthalmology in recent years. As three-dimensional polymeric networks with high water content, hydrogels exhibit excellent biocompatibility, localized targeting capability and sustained-release properties. These characteristics make them ideal carriers for nucleic acid-based drugs, including DNA, RNA and gene editing systems. They can significantly improve the therapeutic efficacy and delivery kinetics of ocular drug administration, demonstrating unique advantages in the special ocular environment. This review systematically examines the current state of hydrogel-based gene therapy research in ophthalmology. First, recent advances in gene therapy are summarized, and the involved gene types and technical strategies are categorized. Second, various categories of hydrogels are outlined, including photo-responsive, temperature-sensitive and pH-responsive hydrogels. Their key characteristics for ocular gene delivery are also summarized. Furthermore, this article focuses on analyzing the design strategies for hydrogel-mediated gene therapy. Key design considerations include gene encapsulation, stability control and release kinetics. In addition, various gene-hydrogel integration strategies are discussed, including covalent conjugation and physical loading methods. Their impacts on transfection efficiency and therapeutic outcomes are also analyzed. Additionally, cutting-edge applications of hydrogel-based gene therapy in treating ocular diseases are systematically introduced, covering both anterior segment and fundus diseases. Finally, the limitations and future development prospects of these gene therapy hydrogel systems in ophthalmic research are discussed.

## Graphical Abstract

**Graphical Abstract:**
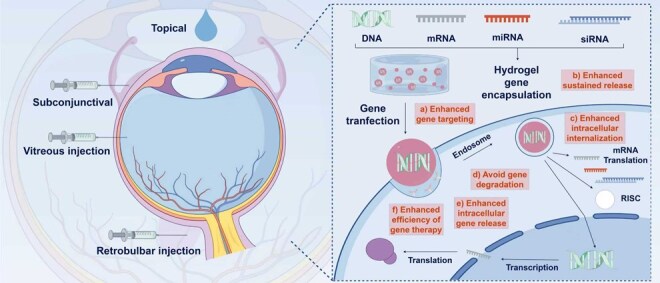

## Introduction

Ophthalmic diseases, due to the unique anatomy of the eye and the critical importance of visual function, have long been a major focus in medical research. Damage to any part of the eye carries the risk of diminishing visual quality [1]. Common ocular conditions, such as keratitis, cataracts, glaucoma, and diabetic retinopathy, can each lead to varying degrees of visual impairment. According to incomplete statistics from the World Health Organization, over 100 million people worldwide suffer from visual impairment [2].

Traditional ophthalmic treatments mainly consist of pharmacotherapy and surgical interventions. Pharmacological treatments are typically administered as eye drops or via other ocular injection routes (e.g. subconjunctival or intravitreal injections), while surgical options include procedures such as laser therapy, vitrectomy and trabeculectomy [3, 4]. Although these approaches can alleviate disease progression to a certain extent, they still face several challenges, largely due to the unique anatomical structures and drug metabolism characteristics of the eye. For example, the corneal barrier helps protect the eye from external irritants, but it also significantly limits effective drug delivery. Additionally, blinking, tear washing and aqueous humor turnover play essential roles in maintaining ocular moisture and reducing frictional damage. However, these physiological processes significantly reduce the bioavailability of traditional eye drops [5]. Although subconjunctival and intravitreal injections can partly overcome the challenges posed by the ocular surface barriers, the invasive nature of repeated procedures introduces risks of local trauma and infection [6, 7]. Moreover, symptomatic treatments, including anti-inflammatory and antioxidant therapies, have shown some progress. However, these treatments remain limited in terms of long-term efficacy, precise intervention and definitive disease resolution. Conversely, modern medicines are often chemically synthesized agents that suffer from drawbacks such as short *in vivo* half-lives, poor tissue distribution and issues of high dependency and resistance with prolonged use [8, 9]. In contrast, gene therapy enables precise and targeted delivery to directly address the root causes of diseases. It also has the potential for long-lasting, “one-and-done” therapeutic effects. Consequently, gene therapy holds vast promise as a future treatment modality for ocular diseases.

Gene therapy is a method that exerts therapeutic or preventive effects by directly targeting genes. Regarded as a revolutionary “one-time treatment” technology, gene therapy is rewriting the history of medicine at an unprecedented pace and ushering in the “Gene Therapy 2.0 era” [10]. Common gene therapy approaches are primarily based on nucleic acids, including DNA, messenger RNA (mRNA), microRNA (miRNA), small interfering RNA (siRNA), antisense oligonucleotides (ASOs) and circular RNA (circRNA) [11]. To date, several gene therapies have received regulatory approval for various diseases. Gene therapy offers innovative therapeutic approaches for both common and rare ocular conditions. However, traditional delivery vectors systems mainly rely on viral vectors, including adenoviruses, adeno-associated viruses and lentiviruses. These viral vectors face several challenges, including limited targeting specificity, high immunogenicity, restricted carrying capacity and transient gene expression. These limitations considerably hinder their broader clinical application [12].

Hydrogels are three-dimensional polymer networks characterized by high water content. Owing to their excellent biocompatibility, loading capacity and tunable physicochemical properties, hydrogels have attracted extensive attention in the field of ophthalmology [13]. Notably, with advancements in engineering technology, biomedical treatment paradigms have gradually shifted toward precision medicine, further expanding the application scope of hydrogels [14]. Hydrogel-mediated gene therapy strategies have been developed and applied to ocular diseases, offering innovative approaches to overcome the limitations of traditional clinical ophthalmic treatments. When combined with gene therapy techniques, hydrogel-based delivery systems offer the following core advantages: (1) precise sustained-release and prolonged therapeutic effects; (2) excellent carrier stability and high transfection efficiency; (3) multimechanism synergistic intervention; (4) minimally invasive procedures with low invasiveness; (5) precise targeted delivery; (6) outstanding biocompatibility, nontoxicity and high safety; (7) superior optical transparency; (8) post-treatment degradability or removability with low immunogenicity; (9) sufficient carrier capacity; (10) scalability for mass production; (11) Tunable mechanical strength [2, 15].

Currently, with advancements in materials science and biomedicine, hydrogel-based gene therapy has been widely developed, significantly accelerating the clinical application of gene therapy. However, comprehensive reviews on hydrogel-mediated gene therapy remain limited. This review provides the first systematic discussion of the gene types and technological approaches involved in gene therapy. In addition, various categories of hydrogel-mediated gene therapy are classified and their design strategies are analyzed. Moreover, this review focuses on the research progress in hydrogel-mediated gene therapy for ophthalmology applications. The advantages and limitations of different therapeutic approaches are discussed and new insights are provided for the future design and application of hydrogel-based gene therapy in ocular treatments.

## Types of therapeutic genes

**Figure 1:**
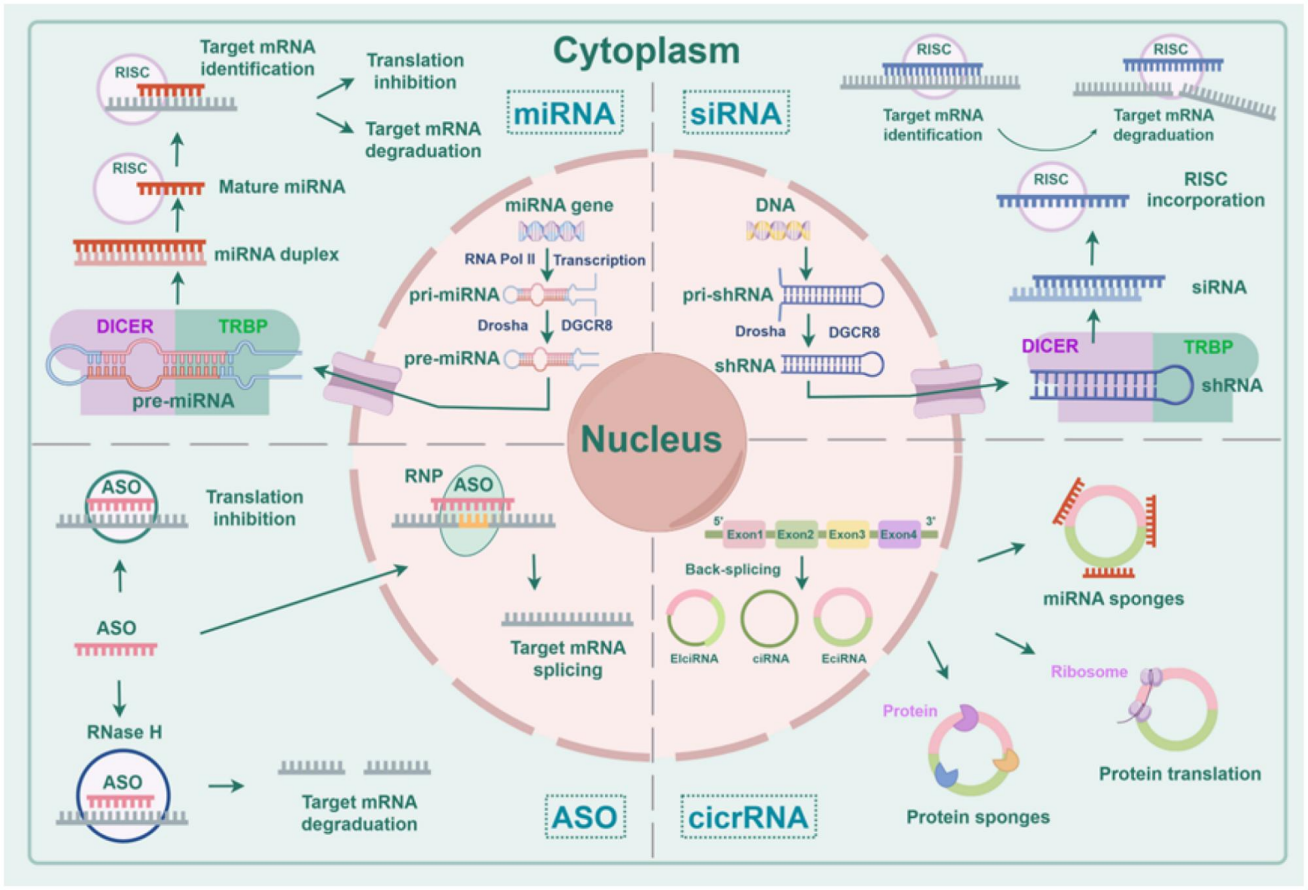
RNA gene therapy classification. Including miRNA, siRNA, circular RNA (circRNA) and ASOs.

The phenomena of life depend on three fundamental molecules: deoxyribonucleic acid (DNA), ribonucleic acid (RNA) and proteins [16, 17]. DNA carries the genetic code; the RNA produced by transcription is utilized to convey genetic information; and the proteins formed through translation are responsible for executing life processes (Figure 1).

### DNA

DNA carries the genetic code and serves as the initial stage in the transmission of genetic information. Errors occurring at the DNA level significantly increase the risk of subsequent errors in RNA transcription and protein synthesis. DNA-based gene therapy can rectify faulty genetic information at its source. By directly introducing exogenous DNA into cells, it is possible to correct genetic defects or enhance specific functions. Genome-editing systems such as CRISPR-Cas9 can be utilized to cut DNA, facilitating the repair of mutated genes. Moreover, by regulating the promoters located upstream of target genes, the spatiotemporal expression of therapeutic genes can be effectively managed.

### RNA

RNA-based gene therapy is a therapeutic approach that employs RNA to treat disease. By modifying RNA, this method can bypass DNA errors and directly affect protein expression, thereby enabling the regulation of specific functions in cellular processes [18]. Based on structure and function, RNA can be categorized into mRNA, noncoding RNAs (including siRNA, miRNA, circRNA among others) and ASOs [19–22].

#### mRNA

mRNA is transcribed from the DNA template of a gene and, despite constituting only a small fraction of the overall RNA pool, encompasses a wide variety of molecules that direct protein synthesis. When introduced into the human body, mRNA leverages the host cell’s protein translation machinery to express its encoded genetic information, thereby producing proteins with the desired functions. Moreover, because mRNA has a relatively short half-life, it is rapidly degraded after completing translation, thereby minimizing its impact on the normal physiological functions of host cells [23].

#### siRNA

siRNA is a double-stranded RNA segment typically 20–25 nucleotides long, usually generated by the Dicer-mediated cleavage of longer double-stranded RNAs. After being incorporated into the ribonucleoprotein RNA-induced silencing complex (RISC), the siRNA forms an activated RISC that specifically binds to target mRNA, thereby causing degradation of the target gene and modulating gene expression [24]. Therefore, specific binding is critical for siRNA to achieve effective gene silencing [25]. Currently, siRNA has been applied in treating various ocular conditions, including retinal diseases, glaucoma, wound healing and neovascularization [26, 27]. However, owing to the relatively low stability of siRNA, it is gradually degraded after entering the cell, limiting the duration of its gene-silencing effects [28]. Based on these considerations, researchers designed short hairpin RNA (shRNA). Similar to siRNA, shRNA is cleaved by the Dicer enzyme to generate siRNA, which subsequently exerts gene-silencing effects [29]. However, because shRNA can form specific secondary structures within the cell, be expressed via DNA vectors and integrate into the host cell genome, it can be continuously transcribed and expressed, thereby generating large quantities of siRNA and achieving long-term silencing of target genes [29, 30]. This also offers new insights for siRNA carrier design. By encapsulating siRNA in hydrogels, it can be protected from degradation and dilution within the body [27, 31].

#### miRNA

miRNA is a noncoding, single-stranded RNA typically around 20–25 nucleotides in length. It is primarily involved in post-transcriptional gene regulation, modulating processes such as RNA processing, modification and translation [32]. Its primary mechanism involves binding to target mRNA, reducing its stability and making it more susceptible to degradation or inhibiting mRNA translation to decrease protein synthesis [33–35]. It is worth noting that a single miRNA can simultaneously regulate multiple target mRNAs. While this increases the potential for miRNAs to modulate multi-gene pathways, it also raises the risk of off-target effects. This suggests that future research could develop hydrogels loaded with miRNAs to enhance their targeting specificity and ensure their stability.

#### CircRNA

CircRNA is a class of noncoding RNA molecules characterized by a covalently closed circular structure, lacking a 5′ cap and a 3′ poly(A) tail. Owing to their covalently bonded circular conformation, circRNA exhibit greater stability compared to linear RNAs, rendering them resistant to exonuclease-mediated degradation. Generated through a process known as “back splicing”, circRNA can be categorized into three types: exonic circular RNAs (EcircRNA), exon-intron circular RNAs (EIcircRNA) and intronic circular RNAs (ciRNA). Precise regulation of this unique biogenesis process offers new avenues for disease diagnosis and treatment in the era of precision medicine. In recent years, an increasing number of functions of circRNA have been uncovered, including translational capability, interaction with mRNAs and interaction with proteins, thereby providing theoretical support for the development of circRNA-based gene therapies targeting ocular diseases.

#### ASO

ASOs are synthetic single-stranded oligonucleotides, typically composed of 15–30 nucleotide sequences. Through precise design, ASOs can hybridize with complementary target RNA sequences, thereby modulating protein expression by interfering with RNA function. ASOs exert their effects via three primary mechanisms. First, ASOs can bind to mRNA and recruit RNase H1, leading to site-specific degradation of the target RNA. This RNase H1-dependent type of ASO is commonly referred to as a gapmer. Second, ASOs can selectively bind to precursor mRNA (pre-mRNA) to modulate alternative splicing, thereby affecting the expression of the target protein. This mechanism enables the exclusion of defective exons from the mature mRNA, allowing the production of functional proteins and offering therapeutic potential for certain genetic disorders. Third, ASOs can interfere with the translation process by binding to the translation initiation site of the target mRNA, thereby inhibiting protein synthesis [36].

## Gene therapy technologies

**Figure 2:**
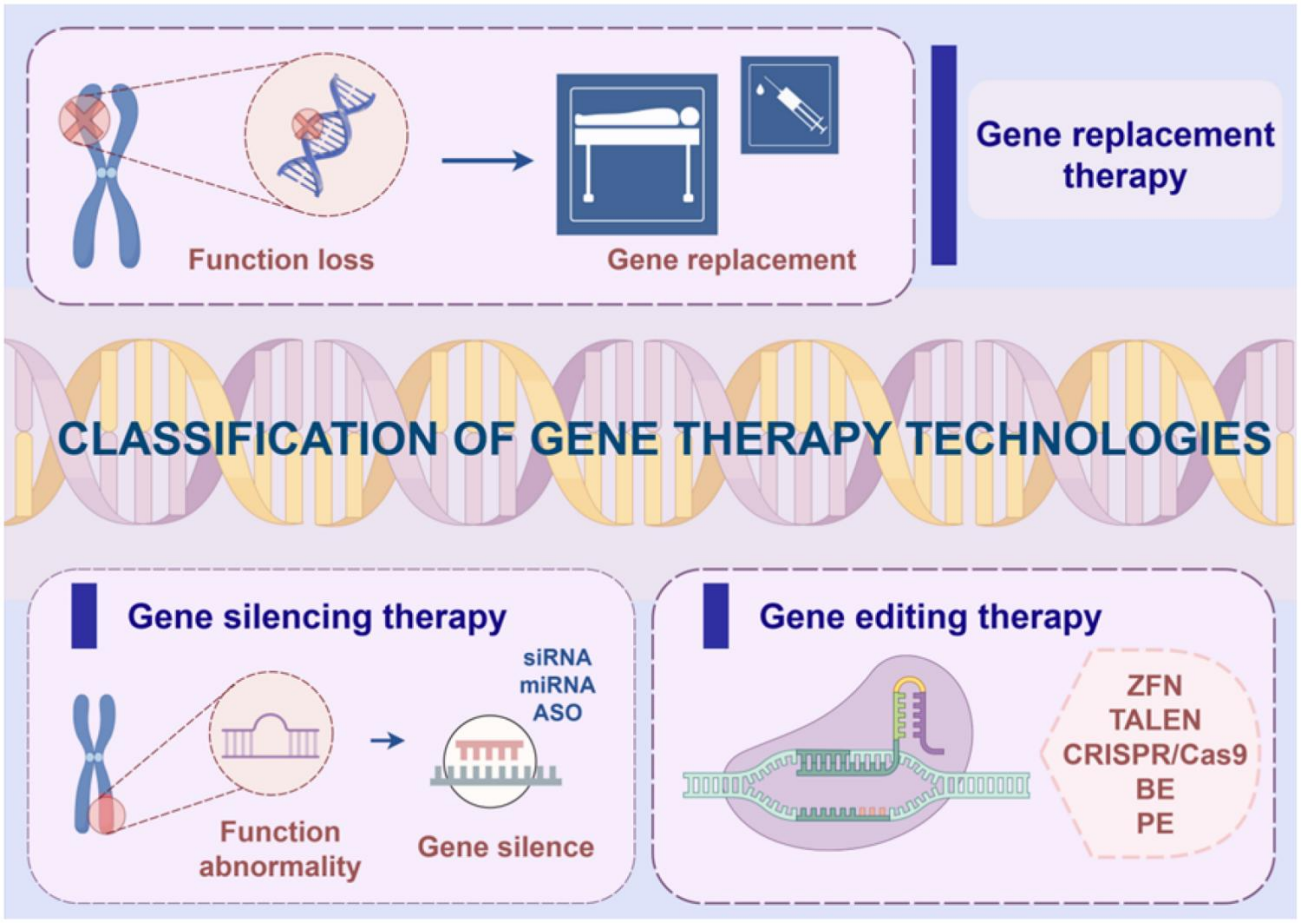
Classification of gene therapy technologies. Including gene replacement therapy, gene silencing therapy and gene editing therapy.

During the 1960s and 1970s, the concept of gene therapy was first introduced [37]. With the tremendous technological advancements in the biomedical field, gene therapy has experienced unprecedented development [38]. In a broad sense, gene therapy refers to the strategy of using genes as “drugs” to intervene in genetic material or regulate gene expression, with the aim of treating diseases through approaches such as gene replacement, gene silencing or gene editing [39–41] (Figure 2).

### Gene replacement

Gene replacement therapy (GRT) is one of the primary methods in the field of gene therapy. Its core strategy involves delivering or supplementing a normal gene to restore normal cellular function, thereby compensating for the deficiencies of a defective gene [42]. By directly supplementing the missing functional gene to achieve functional compensation, GRT can target the underlying genetic cause, enabling precise localization and localized delivery. GRT has achieved significant breakthroughs in the treatment of ophthalmic diseases and is particularly well-suited for ocular disorders caused by monogenic mutations. Leber congenital amaurosis type 2 (LCA2) is an inherited retinal disorder caused by mutations in the RPE65 gene. The RPE65 gene plays a critical role in the visual cycle by participating in the function of the retinal pigment epithelium, facilitating the conversion of all-trans-retinal to 11-cis-retinal, which is essential for the proper synthesis and regeneration of rhodopsin. The primary clinical manifestations of LCA2 include night blindness and progressive visual impairment. Currently, gene therapy is the only effective treatment option for LCA2. By supplementing functional RPE65, this approach corrects the underlying genetic deficiency, thereby achieving therapeutic benefits. Adeno-associated virus (AAV) vectors are the most commonly employed delivery systems in GRT due to their favorable safety profile [43]. Different AAV serotypes exhibit distinct tissue tropisms. AAV2, AAV8 and AAV9 are commonly used for the treatment of retinal diseases, while AAV5 is a preferred choice for corneal delivery via topical administration. In 2017, the U.S. Food and Drug Administration (FDA) approved voretigene neparvovec-rzyl (VN) (marketed as Luxturna), the first gene therapy for ophthalmic use worldwide. This therapy utilizes AAV2 as a vector to deliver a functional RPE65 gene, thereby compensating for the deficiency associated with RPE65-related disorders. To date, AAV-based therapies have been applied to a range of diseases and have demonstrated promising progress. However, due to its limited cargo capacity (<5 Kb), AAV is unable to deliver larger genes, which significantly hinders the advancement of GRT [44–46]. The widespread prevalence of anti-AAV neutralizing antibodies in the population also presents certain clinical challenges for AAV GRT [47, 48]. Additionally, the presence of dominant-negative effects presents a significant limitation for GRT, making it challenging to cure dominant genetic disorders [49, 50]. Integrating hydrogel-based delivery systems can optimize GRT by enabling the delivery of larger genes and enhancing transfection efficiency. Additionally, these systems can concurrently deliver related RNAs (such as siRNA or shRNA) or other genes, thereby facilitating the treatment of dominant genetic disorders or diseases caused by multiple gene mutations.

### Gene silencing

Gene silencing (GS) therapy exerts its therapeutic effects primarily by inhibiting the expression of aberrant genes. It holds significant potential in the field of ophthalmology, particularly for the treatment of ocular diseases driven by genetic mutations, dysregulated gene expression or inflammation, such as retinitis pigmentosa (RP), neovascular age-related macular degeneration (n-AMD) and dry eye disease [51]. Common GS techniques include RNA interference (siRNA or shRNA), ASOs and CRISPR interference (CRISPRi) [52]. Drugs derived from this technology are commonly referred to as small nucleic acid therapeutics, specifically RNA interference (RNAi)-based drugs. In 1998, FDA approved the world’s first ASOs drug, Vitravene, for the treatment of cytomegalovirus (CMV) retinitis in the eye. In 2004, Bevasiranib, a siRNA drug targeting vascular endothelial growth factor (VEGF), entered clinical trials for the treatment of n-AMD. GS can rapidly inhibit pathogenic genes; however, under repeated pathological stimulation, these genes may be reactivated, necessitating multiple administrations of gene silencing agents. Hydrogel-mediated GS techniques offer sustained release properties, which prolong the duration of GS, thereby reducing the frequency of dosing and improving patient compliance. One of the major challenges in the development of small nucleic acid therapeutics lies in ensuring their precise delivery to target cells after administration into the human body. An efficient and safe delivery system can significantly enhance the specificity of drug targeting to desired cells. Hydrogel-based delivery systems with tailored specificity offer a promising strategy for achieving efficient targeted delivery. Moreover, the hydrogel delivery system can protect nucleic acid drugs from degradation by nucleases, thereby prolonging their half-life *in vivo*.

### Gene editing

**Figure 3:**
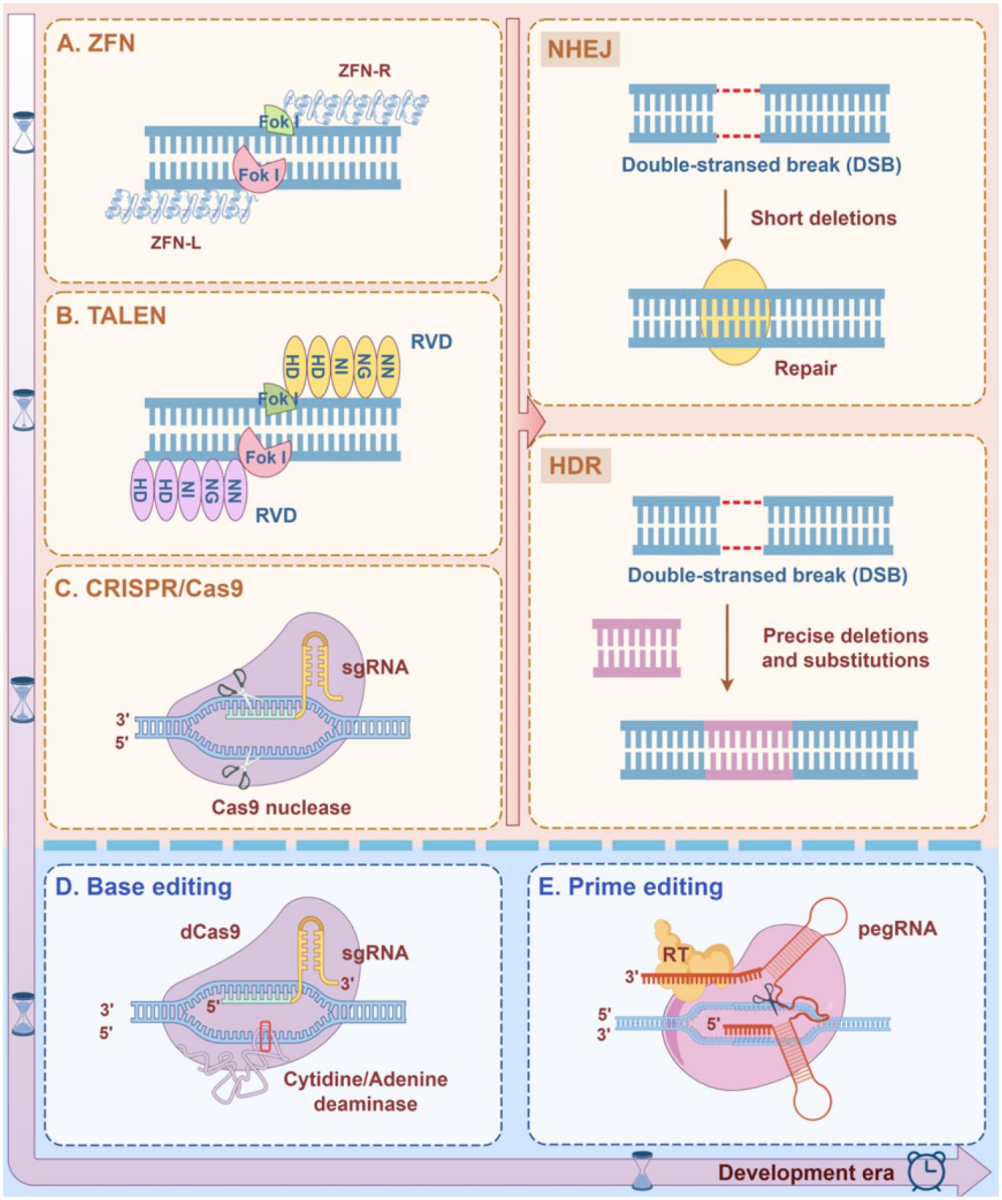
Classification of gene editing therapies and their evolution. Including (**A**) first-generation ZFN, (**B**) second-generation TALEN, (**C**) third-generation CRISPR/Cas9, as well as the newest approaches in (**D**) BE and (**E**) PE.

Gene editing is a genetic engineering technique that enables precise modifications and alterations to targeted genomic sequences. With the rapid advancement of biotechnology, gene editing therapies have emerged as a prominent field in modern biological research and applications. Following three generations of technological evolution, gene editing methods now include first-generation zinc finger nucleases (ZFN), second-generation transcription activator-like effector nucleases (TALEN), third-generation CRISPR/Cas9, as well as the newest approaches in base editing (BE) and prime editing (PE) [53–56] (Figure 3). Currently, CRISPR/Cas9 is widely used due to its simplicity and high efficiency, making it one of the most promising gene editing tools [57]. The process primarily involves three steps: targeting the specific DNA sequence, inducing a DNA double-strand break (DSB), and introducing a knockout or knock-in during the repair process. The basic principle relies on guide RNA (gRNA) that pairs complementarily with the target DNA, directing the Cas9 nuclease to cleave the intended DNA sequence, thereby triggering the cell’s intrinsic repair mechanisms to achieve gene knockout, insertion or replacement [58, 59]. DNA’s intrinsic repair mechanisms primarily encompass two pathways. One is nonhomologous end joining (NHEJ), which can induce frameshift mutations at the repair site, leading to gene inactivation and thus, achieving targeted gene knockout; the other is homology-directed repair (HDR), which utilizes an exogenous DNA template to facilitate specific gene insertion or replacement [60–62]. Gene editing technology has widespread applications in ophthalmic diseases. Zhen *et al.* utilized CRISPR/Cas9 gene editing technology to target and edit the VEGFA gene to prevent corneal neovascularization (CNV) [63].

In the overall CRISPR/Cas9 process, the most critical step is targeting the desired sequence. This implies that researchers should improve the specificity of CRISPR/Cas9 by enhancing the base-pair complementarity between the gRNA and the target genomic sequence [64]. When sequences that are complementary to the gRNA occur at nontarget sites, off-target effects arise, potentially disrupting the normal expression of genes [65]. This also suggests that researchers could further investigate methods to enhance gRNA specificity. The synergistic application of hydrogels with CRISPR/Cas9 offers one strategy to address these challenges. The three-dimensional polymer network of hydrogels can encapsulate CRISPR/Cas9 complexes and through sustained release, protect gRNA from nuclease degradation, thereby ensuring precise base-pair complementarity with the target genomic sequence. Additionally, covalent attachment of cell-specific ligands to the hydrogel network can augment its selective affinity for target cells, ensuring that CRISPR/Cas9 complexes preferentially accumulate near the intended cells, which reduces off-target effects. Furthermore, BE and PE are next-generation technologies developed on the CRISPR/Cas9 platform that overcome the traditional requirement for DSBs, enhance genomic stability and further minimize off-target effects [66–68]. Fu *et al.* [69] employed PE gene editing technology to perform *in vivo* editing of the PDE6B gene Y347X mutation in a mouse model of RP. They successfully restored PDE6B protein expression, which protected rod cell function, offering a new strategy for gene therapy in RP. Looking ahead, CRISPR/Cas9 presents an effective therapeutic approach for complex mutational diseases, while BE and PE offer even more potent strategies for treating single-base point mutations and intricate mutational disorders [70]. In the field of ophthalmology, the integration of hydrogel-targeted delivery systems will further advance the precise treatment of ocular diseases.

As a representative of next-generation therapeutics, gene therapy constitutes a major breakthrough in the life sciences. Nonetheless, it faces challenges related to safety, delivery efficiency and long-term efficacy. Notably, the eye possesses unique anatomical and physiological characteristics, including compartmentalization and immune privilege, which confer significant advantages for localized gene delivery. These features reduce the likelihood of systemic inflammatory responses. Moreover, the small volume of the eye allows for the use of lower doses of viral vectors, further mitigating immunogenicity. Together, these attributes establish the eye as an ideal target organ for gene therapy, providing a strong rationale for its application in the treatment of inherited, degenerative and inflammatory ocular diseases.

Gene therapy represents a cutting-edge strategy for the treatment of ocular diseases and primarily includes three approaches: gene replacement, GS and gene editing. Each of these modalities is suited to different types of eyes disorder and possesses distinct advantages and limitations. Gene replacement is most applicable to diseases caused by loss-of-function mutations, such as LCA2. GS is better suited for conditions driven by aberrant gene expression, such as choroidal neovascularization; however, it is limited by a relatively short duration of action. Gene editing offers the potential to correct a broader range of genetic variants and can permanently repair pathogenic mutations, thereby compensating for some of the shortcomings of GS. In summary, gene therapy has achieved revolutionary breakthroughs in ophthalmic diseases, especially in inherited retinal diseases (IRDs), and is actively expanding to acquired eye diseases such as neovascular (wet) age-related macular degeneration (AMD). Although gene therapy still faces challenges such as immune response, disease complexity and long-term safety concerns, continuous advancements in vector technologies and gene editing techniques are paving the way for gene therapy to become a promising “one-time” curative option for patients with ocular diseases in the future.

## Hydrogel-mediated gene therapy

### Advantages in hydrogel-mediated gene therapy

Gene therapy is a precise treatment modality that utilizes genes or gene editing systems to modulate gene expression within the body. However, the direct delivery of naked DNA or RNA presents numerous challenges, such as low nucleic acid stability, susceptibility to degradation by endogenous nucleases, poor tissue penetration and limited targeting efficiency [71]. Consequently, delivery vectors play a critical role in safeguarding and transporting these genetic materials. Vector-based delivery not only protects genes from nuclease-mediated degradation but also enhances penetration and targeting through vector modifications such as charge interactions or ligand conjugation.

Traditional vectors for gene therapy include viral and nonviral vectors. Common viral vectors include AAV, adenovirus and lentivirus [72]. These viral vectors exploit the natural infectious properties of viruses to efficiently enter host cells, thereby enabling high-efficiency and stable expression of target genes. Although viral vectors exhibit strong gene delivery capabilities, such as high transfection efficiency and prolonged expression, their use also entails certain safety risks, including severe immune responses and potential viral reactivation [44, 45]. In contrast, nonviral vectors primarily rely on physical or chemical methods such as microinjection, electroporation and plasmid delivery to introduce nucleic acids or gene editing systems into cells. This approach enhances the precision of gene therapy while mitigating the immunogenic risks associated with viral vectors; however, nonviral delivery methods typically suffer from lower transfection efficiency and shorter duration of action [73].

However, owing to advantages such as high safety, large gene-loading capacity and strong flexibility, nonviral vector–mediated gene therapy has become an important direction in the field of gene therapy. At present, nonviral vectors mainly include liposomes, polymeric materials, inorganic nanoparticles and hydrogels. By selecting appropriate cationic lipids, gene-loaded liposomes can be formed through self-assembly driven by electrostatic interactions [74]. This delivery strategy is one of the most technically mature and widely used nonviral vectors, exhibiting relatively high transfection efficiency and gene-editing capability [75]. Nevertheless, this approach has difficulty achieving long-term local retention and the presence of cationic lipids leads to increased cytotoxicity and reduced biocompatibility. Polymeric carriers mainly include polyethyleneimine (PEI), poly(lactic-co-glycolic acid) (PLGA) and PAMAM dendrimers. These systems provide better protection for nucleic acids and polymeric materials also allow certain chemical modification functions. However, their biocompatibility is relatively poor, and the degradation products carry a higher risk of inducing inflammatory responses. Inorganic nanoparticles, such as gold nanoparticles and silica nanoparticles, have also attracted increasing attention in recent years. They possess stable structures and can be combined with auxiliary therapeutic strategies such as photothermal effects, enabling integrated imaging and therapy, as well as simultaneous gene delivery and thermal ablation of target cells to achieve synergistic treatment [76, 77]. Nevertheless, the risk of long-term accumulation of inorganic nanoparticles remains to be thoroughly investigated, and their clinical safety is still highly controversial.

With the interdisciplinary advances between materials science and gene therapy, hydrogels have gradually emerged as a novel nonviral delivery strategy for gene therapy. Hydrogels are three-dimensional polymer networks with high water content that have historically garnered attention in drug delivery and tissue engineering [78, 79]. In drug delivery, systems are required to offer controlled release and targeted delivery, while tissue engineering demands superior biocompatibility [80–82]. Hydrogels effectively address the shortcomings of traditional drug delivery systems by modulating release rates and improving targeting capabilities through stimuli-responsive mechanisms. This enables the release of therapeutic agents near target cells, thereby minimizing uneven distribution and reducing toxicity. In the context of gene therapy, hydrogels leverage these advantages to localize gene transfer at the target site, thereby mitigating off-target effects [83]. Therefore, compared with the nonviral delivery strategies mentioned above, hydrogels are not single delivery particles but rather a platform for localized regulation. Compared with the one-time delivery mode of other nonviral vectors, the core advantage of hydrogels lies in their sustained, slow and controllable gene release. This release profile prolongs the *in vivo* activity of gene therapeutics and increases their effective concentration. It also reduces dosing frequency and improves compliance. In addition, as a locally retained sustained-release platform, hydrogel carriers exhibit a lower risk of entering the systemic circulation. This property limits gene distribution to nontarget tissues and thereby reduces systemic immune side effects. Such local retention is particularly suitable for organs with semi-enclosed structures, such as the eye, and further enhances the local sustained-release performance of hydrogels.

This lays the theoretical foundation for the application of hydrogel-mediated gene therapy in the eye. Due to the complex structure of the ocular surface, which includes unique barrier properties and drug metabolism pathways, free nucleic acids have difficulty penetrating and being delivered to target tissues. Hydrogel delivery systems can closely adhere to the ocular surface, prolonging contact time and, to some extent, overcoming the limitations of traditional eye drops, such as short ocular retention time and difficulty in localized delivery, thus, improving gene delivery efficiency. Additionally, the excellent tissue compatibility of hydrogels reduces local irritation and immunogenicity, establishing a foundation for the long-term treatment of chronic eye diseases. At the same time, the encapsulation effect of hydrogels helps maintain the biological activity of genes and protects nucleic acid molecules from degradation by nucleases in the body, thereby enhancing their bioavailability [82, 84]. Designing responsive hydrogels sensitive to factors such as temperature and pH can enable on-demand release in response to the ocular microenvironment, further controlling the drug release rate and reducing the side effects caused by gene burst release. Moreover, the strategy of embedding nanovesicles within hydrogels allows for the synergistic regulation of ocular surface adhesion and penetration. By utilizing nanovesicle-hybridized hydrogels for gene therapy, low-frequency, low-dose, minimally invasive and highly penetrative treatments for retinal diseases can be achieved. The design strategy of injectable hydrogels also meets the needs for vitreous injections in the treatment of retinal or choroidal diseases.

Hydrogels, with their controlled sustained release properties, excellent biocompatibility and precise delivery capabilities, enhance the stability of genes or gene editing systems, avoiding the immunogenic risks associated with traditional viral vectors, reducing the side effects related to systemic drug administration, minimizing off-target effects and decreasing the need for multiple administrations, thereby improving patient compliance. This strategy effectively addresses the core requirements of ophthalmic gene therapy for safety, sustainability and high precision, and is highly aligned with the evolving trends of personalized and precision medicine. In the future, with the integration of nanotechnology, intelligent responsiveness and personalized treatment strategies, hydrogels are expected to become an important platform for advancing the clinical translation of gene therapy in ophthalmology [85].

### Classification of hydrogels for mediating gene therapy

Hydrogels can be categorized based on their origin into natural and synthetic types. Generally, natural hydrogels exhibit superior biocompatibility, while synthetic hydrogels offer enhanced physicochemical tunability [13, 86, 87]. The design of composite hydrogels that integrate natural and synthetic elements can synergistically combine the benefits of both, thereby maximizing functionality. In recent years, driven by continuous advancements in materials science, researchers have developed a broader array of smart hydrogels capable of responding to specific physical stimuli (e.g. light, temperature) or chemical stimuli (e.g. pH, reactive oxygen species [ROS]), which facilitates either the release of encapsulated agents or the degradation of the hydrogel network [88–91] (Table 1). By incorporating appropriate intelligent systems and leveraging the adjustable physicochemical properties of hydrogels, novel hydrogel systems for mediating gene therapy have been engineered to achieve localized adhesion, targeted delivery, barrier penetration and controlled sustained release, thereby providing an innovative alternative to conventional delivery strategies.

**Table 1:** Comparison of responsive hydrogels classifications.

Types	Response mechanisms	Core materials	Trigger conditions
Light-responsive	Free radical addition; o-Nitrobenzyl esters, coumarin esters	GelMA	Specific wavelengths: ultraviolet light (365 nm) or blue light (405 nm)
Thermo-responsive	LCST, UCST	Chitosan	Specific temperature: body heat or localized heat therapy (>37 °C)
pH-responsive	Protonization or deprotonation	HNP hydrogel	Specific pH: inflammatory or tumor microenvironment (pH < 7.0)
ROS-responsive	oxidative cleavage (-S-, -Se-), Thiol response (-S-S-)	CE-MN	Inflammatory microenvironment

#### Light-responsive hydrogels

Based on their mechanisms of response to light, light-responsive hydrogels can be categorized into several types, including photopolymerizable hydrogels, photo-degradable hydrogels and photoisomerizable hydrogels [92–94]. Photopolymerizable hydrogels exhibit crosslinking under light exposure, leading to a phase transition from a liquid to a solid state [95]. This property allows researchers to precisely control the spatial and temporal aspects of hydrogel activation. Due to the convenience of light, photocurable hydrogels have found extensive applications in ophthalmology [96–98]. For instance, Zhao *et al.* [99] developed a photocurable double-network hydrogel based on gelatin methacrylate (GelMA) and oxidized dextran (ODex), which adheres seamlessly to the cornea and promotes corneal epithelial regeneration. This suggests that future research could focus on employing photopolymerizable hydrogels to mediate gene therapy for corneal repair.

Additionally, because light can penetrate from the anterior segment of the eye to the posterior segment, gene therapy mediated by photocurable hydrogels has shown promising prospects in treating posterior segment diseases. In contrast, photo-degradable hydrogels enable the release of encapsulated substances through the disruption of the polymer network induced by light exposure. The key to this mechanism is the presence of photolabile groups such as o-nitrobenzyl ester and coumarin ester which decompose or cleave in response to specific light sources [100–102]. This is fundamentally different from photopolymerizable hydrogels, which form networks under light exposure and are more conducive to the sustained release of genes or gene-editing systems; photo-degradable hydrogels, conversely, are better suited for precise release due to their light-induced network disruption. While both photopolymerizable and photo-degradable hydrogels exhibit irreversible responses to light, photoisomerizable hydrogels undergo reversible molecular conformational changes. This reversibility offers potential applications in controlled, reversible drug release. Despite the different mechanisms underlying their light responses, all these hydrogels share the common feature that adjusting parameters such as light wavelength, intensity, duration or location allows for remote modulation of their properties.

#### Thermo-responsive hydrogels

Thermo-responsive hydrogels undergo either reversible or irreversible phase transitions from a liquid to a solid state in response to temperature changes [103]. Depending on the temperature at which the reaction occurs, they can be categorized into thermosensitive hydrogels and cold-sensitive hydrogels [14]. Thermosensitive hydrogels exist in a fluid state at low or room temperatures and transition to a gel state upon heating or under body temperature conditions, while cold-sensitive hydrogels exhibit the opposite behavior [103]. Due to their ability to gel at body temperature, thermosensitive hydrogels show significant promise and therapeutic potential. When mixed with genes or gene delivery systems under ambient conditions and subsequently administered *in vivo*, these hydrogels undergo phase transition at body temperature [104]. Based on this property, thermosensitive hydrogels can serve not only as drug reservoirs for genes or gene delivery systems but also as scaffolds for cell growth [105]. Cold-sensitive hydrogels, in contrast, remain in a liquid state at room temperature and convert to a gel state upon heating or at body temperature [14]. For instance, Tang *et al.* developed a novel approach for treating corneal diseases by combining exosomes derived from induced pluripotent stem cell-derived mesenchymal stem cells (iPSC-MSCs) with a thermosensitive chitosan hydrogel (CHI hydrogel). The CHI hydrogel facilitates sustained release of miR-423-5p, thereby promoting effective corneal repair and reducing scar formation [106]. This suggests that future research could further expand the application of thermo-responsive hydrogels in mediating gene therapy in ophthalmology.

#### pH-responsive hydrogels

pH-responsive hydrogels are intelligent systems that undergo structural or morphological changes in response to variations in environmental pH. The core mechanism behind their pH responsiveness lies in pH-sensitive chemical bonds, which act as smart switches by undergoing protonation or deprotonation under specific pH conditions, thereby altering the polymeric structure of the hydrogel [107, 108]. Examples of such bonds include imine, amide and ketal bonds, which remain relatively stable under neutral or alkaline conditions but dissociate in acidic environments [109, 110]. This property renders pH-responsive hydrogels highly promising for applications in cancer therapy and wound infection management. During cancer progression or bacterial infections, the metabolic microenvironment shifts, leading to the accumulation of acidic substances (e.g. lactate, carbonate) that lower the local pH; this in turn triggers the disintegration of the hydrogel through the cleavage of pH-sensitive bonds, resulting in the release of its internal payload [107, 109]. Additionally, Khaled and colleagues synthesized a pH-responsive hydrogel based on nanostructured inorganic silica cores and cationic poly(2-(diethylamino)ethyl methacrylate). This system can modulate the loading or release of encapsulated siRNA according to the environmental pH, effectively preventing the enzymatic degradation of siRNA [111].

While current research has largely focused on pH-responsive hydrogel drug delivery systems for ocular diseases, their application in gene therapy for such conditions remains limited. By exploiting the variable pH of the ocular environment, these hydrogels can achieve controlled release of therapeutic genes, thereby facilitating targeted and sustained treatment of ocular diseases. This pH-responsive hydrogel-based gene therapy approach holds significant promise and may pave the way for broader clinical applications in the future.

#### Reactive oxygen species responsive hydrogels

An increasing body of evidence suggests that the pathological conditions of many ocular diseases are associated with ROS [112]. Consequently, the development of ROS-responsive hydrogel systems holds tremendous potential. By utilizing ROS-responsive materials, hydrogels can be engineered to degrade their chemical bonds under oxidative conditions reflective of an imbalanced microenvironment, leading to the disintegration of the polymer network and enabling targeted drug delivery [113]. Dry eye disease one of the most prevalent ocular surface disorders—has been clearly linked to ROS-related mechanisms. Mu *et al.* [114] developed a ROS-responsive microneedle patch (CE-MN) capable of penetrating the periorbital skin. This patch modulates drug release in response to ROS, thereby achieving minimally invasive delivery to the lacrimal gland region and ensuring localized drug accumulation. Moreover, the use of an AAV vector to supplement the aquaporin-1 (AQP-1) gene in a mouse model of Sjögren’s syndrome has been shown to restore tear secretion [115]. Although the application of ROS-responsive hydrogels in gene therapy for ocular diseases is still in its early stages, these promising data provide a solid theoretical basis for future development of ROS-responsive hydrogel-based gene therapies.

## Design strategies for hydrogel-mediated gene therapy

Direct therapeutic application of genes typically faces challenges such as low stability and limited penetrability, which contribute to suboptimal gene utilization [116]. Consequently, loading genes onto carriers becomes essential. Hydrogels, with their excellent biocompatibility, highly hydrated three-dimensional polymer networks and tunable physicochemical properties, represent promising carriers for gene delivery.

One common approach involves directly mixing the gene with a hydrogel precursor solution. As the precursor undergoes a phase transition under suitable conditions, the gene becomes encapsulated within the three-dimensional network. Although this method is straightforward and efficient, it often results in relatively low gene release efficiency [117]. The release rate of the gene from the hydrogel is primarily governed by passive diffusion driven by concentration gradients and the crosslinking density of the network. Enhancing the stimulus-responsiveness of the hydrogel can improve its degradability, thereby accelerating the gene release rate. Additionally, maintaining the stability of the gene during direct mixing is a critical consideration; incorporating nucleic acid protectants such as trehalose can, to some extent, prevent nucleic acid degradation [118]. Pre-encapsulating the gene within nanocarriers and subsequently dispersing these nanoparticles into the hydrogel represents another direct gene-loading strategy [119]. In this approach, the hydrogel functions primarily as a responsive matrix, modulating the release of nanoparticles in reaction to external stimuli and thereby enhancing sustained release and adhesion. Meanwhile, the nanoparticles serve to protect the gene from degradation and facilitate targeted delivery [120, 121].

### The interaction mechanism between hydrogels and genes

Hydrogels, as three-dimensional networks, are primarily formed through physical and chemical crosslinking [122]. Physical crosslinking, also known as noncovalent crosslinking, offers the advantages of mild conditions and high reversibility, although it typically exhibits relatively low mechanical strength. Common physical crosslinking mechanisms include electrostatic interactions, hydrogen bonding and hydrophobic interactions [123, 124]. Due to their high reversibility, physically crosslinked hydrogel drug delivery systems often display characteristics of short-term drug release. In contrast, chemically crosslinked hydrogels demonstrate different mechanical properties; they offer higher stability and superior mechanical performance. Depending on their reversibility, chemical crosslinking can be categorized into covalent crosslinking and dynamic covalent crosslinking. Common covalent crosslinking methods include free radical polymerization, click chemistry and Schiff base reactions [124]. Owing to their enhanced stability, covalent crosslinking is particularly suitable for tissue engineering scaffolds. Dynamic covalent crosslinking, which includes disulfide and imine bonds [125], combines the stability of covalent bonds with the reversibility of noncovalent bonds, endowing hydrogels with self-healing and environmental responsiveness [126], which laying the groundwork for their application in smart drug delivery systems.

Genes (e.g. plasmid DNA, siRNA or mRNA) can interact with hydrogels through physical crosslinking. Electrostatic interactions refer to the attractive or repulsive forces between two entities that arise from the presence of electric charges. Objects bearing opposite charges will experience mutually attractive electrostatic forces. Due to the acidic phosphate groups within nucleic acid molecules, nucleic acids typically bear a negative charge in alkaline solutions. Therefore, genes generally cannot directly fuse with the cell membrane *in vivo* and must rely on delivery carriers to be transported into cells. Except for cationic hydrogels composed of PEI, chitosan or quaternary ammonium–modified polymers, most hydrogels are typically neutral or negatively charged [127]. To enhance gene delivery efficiency, surface modification and charge regulation of hydrogels are critical. By introducing positively charged functional groups, such as amino groups (–NH_2_) and quaternary ammonium groups (–${\text{NR}}_{3}^{+}$), electrostatic interactions between hydrogels and negatively charged genes can be promoted. By imparting positive charges to hydrogels, electrostatic adsorption between the hydrogel matrix and negatively charged genes can be exploited, facilitating the binding of hydrogels with negatively charged DNA or RNA to form stable gene–hydrogel complexes [128, 129]. Hydrogen bonds formed between the hydrogel and nucleic acid bases can also enhance the loading of nucleic acids onto the hydrogel [130, 131]. This gene-hydrogel composite approach not only effectively stabilizes gene molecules, preventing their degradation by nucleases in the body, but also promotes cellular uptake of the genes, enhancing endocytosis. Furthermore, by introducing molecules with specific targeting functions (such as antibodies or peptide sequences) on the surface of the hydrogel, the targeting efficiency of gene delivery can be further improved, thereby reducing potential toxicity to nontarget cells. Although electrostatic interactions are highly effective in gene therapy, they also pose risks of cytotoxicity and immune reactions. Therefore, optimizing the biocompatibility of electrostatic interactions is crucial. By adjusting the charge density of the hydrogel and appropriately introducing neutral or negatively charged groups during surface modification, the excessive charge of cationic groups can be partially shielded, reducing negative effects on cells. Additionally, introducing degradable segments (such as ester bonds and disulfide bonds) to regulate the degradation rate is an important strategy for enhancing the biocompatibility of the hydrogel. In summary, during gene therapy, the strategy of modifying the hydrogel surface, combining it with cationic complexes and using receptor-mediated mechanisms on cell membranes to introduce targeting ligands on the hydrogel surface can enhance the endocytosis of the gene-hydrogel composite by cells, thereby improving targeting efficiency while delivering genes. This approach can effectively address the charge repulsion challenge during cellular uptake of genes [132]. By further regulating the charge density and incorporating biodegradable design, cytotoxicity can be reduced and biocompatibility can be enhanced, which will further increase the potential of hydrogel-based gene therapy for applications in other biomedical fields.

Furthermore, genes can also be conjugated to hydrogels via the aforementioned chemical crosslinking. Covalent coupling of hydrogels with genes is an important strategy in gene delivery. By linking hydrogels and gene molecules through covalent chemical bonds, stable gene loading and controlled, precise release can be achieved [133]. Compared with simple physical adsorption, this approach offers greater stability and is less prone to dissociation or loss of function *in vivo*. The fundamental structural unit of DNA is the deoxynucleotide, which comprises a base, deoxyribose and phosphate. In contrast, the basic building block of RNA is the nucleotide, consisting of a base, ribose and phosphate [134]. From the perspective of chemical crosslinking feasibility, both components contain multiple reactive functional groups. For example, nucleobases such as adenine, guanine and cytosine contain amino groups (–NH_2_); cytosine, guanine and uracil contain carbonyl groups (C = O); and the hydroxyl group (–OH) on the 2′-carbon of ribose can also serve as a modification site [135, 136]. These features provide ample opportunities for the chemical modification of nucleic acid molecules. Therefore, the key to covalent coupling between hydrogels and genes lies in the selection of appropriate chemical reactions and functional groups. For instance, amine groups can be introduced at the termini of hydrogels through chemical modification, allowing them to react with amino groups on gene molecules to achieve covalent coupling between the gene and the hydrogel. Alternatively, esterification reactions can occur between amino groups on gene molecules and carboxyl groups on the hydrogel; activated ester reagents such as EDC and NHS can accelerate this process, and the coupling density and efficiency can be controlled by adjusting the reaction conditions [137]. In addition, introducing suitable reactive groups on both the hydrogel and the gene not only increases the available crosslinking sites but also enables integration with other physical interactions, such as photo-crosslinking or thermoresponsive mechanisms. This provides additional levels of control over hydrogel–gene conjugation. Such multidimensional crosslinking strategies not only enhance the stability of hydrogel–gene composites but also endow them with greater tunability, further expanding the application scope of hydrogel-based gene therapy. It is worth noting that although covalent conjugation generally results in higher encapsulation efficiency and reduced gene dissociation and leakage, the crosslinking reactions involved may, to some extent, disrupt the secondary structure of nucleic acids. In addition, precise control over the cleavage of covalent linkages at appropriate sites to enable efficient gene release remains a critical design challenge [138]. Therefore, optimizing reaction conditions to maximally preserve nucleic acid secondary structures while improving the controllability of gene release from hydrogel–gene complexes, thereby enhancing transfection efficiency, represents an important direction for future research.

By designing smart, responsive hydrogels, it is possible to achieve efficient, targeted and controllable sustained gene delivery by leveraging the hydrogel’s tunable physicochemical properties. This offers a novel alternative for gene therapy carriers. However, it is important to note that different gene therapy modalities have distinct limitations, which in turn impose varied requirements on hydrogel design. For example, mRNA suffers from poor stability and often necessitates multiple administrations. Thus, an mRNA delivery system based on hydrogels must be engineered for high stability and prolonged release. In such a system, mRNA is loaded into a specially designed hydrogel where the three-dimensional polymer network effectively shields it from nucleases *in vivo*, thereby slowing degradation, while the hydrogel’s sustained release profile maintains continuous mRNA delivery to overcome the limitation of transient expression requiring frequent dosing. Similarly, the CRISPR-Cas9 system carries a risk of off-target effects. A common strategy to mitigate this risk in hydrogel-based delivery systems is the incorporation of targeting peptides, which can significantly reduce off-target activity by concentrating the system near the intended receptor. Moreover, the employment of photosensitive or thermosensitive hydrogels further enables precise spatiotemporal control of release, enhancing the likelihood of accurate matching between the gRNA and its target sequence. In summary, given the distinct mechanisms and inherent challenges of different gene therapy approaches, the integration of specifically designed hydrogel delivery systems holds promise for overcoming traditional limitations and further advancing the field of gene therapy [73].

## Hydrogel-mediated gene therapy in ophthalmology

The eye possesses unique barrier structures that, while limiting drug bioavailability to some extent, also create opportunities for gene therapy strategies. Specifically, the presence of enclosed compartments such as the subconjunctival and vitreous chambers allows for local injections, thereby reducing systemic exposure and enhancing safety. Furthermore, the blood-ocular barrier provides a low-immunogenic environment within intraocular tissues, which diminishes the potential immune reactions elicited by viral vectors [139].

Considering that different diseases have distinct pathological mechanisms; gene therapy hydrogel systems must be tailored to meet diverse functional and physicochemical requirements. For conditions such as corneal injuries, corneal inflammation and CNV, these hydrogels are designed to provide adhesion and sustained-release properties, thereby reducing the need for frequent administrations. In the case of retinal detachment and vitreous-related disorders, the hydrogels are engineered to deliver the necessary mechanical strength. Meanwhile, for retinal neovascular diseases, the hydrogel systems aim to offer synergistic therapeutic effects alongside controlled release to promote effective disease resolution. Moreover, due to significant differences in pathogenic genes and disease mechanisms among various ocular disorders, the most appropriate gene therapy strategies vary accordingly. Hereditary corneal dystrophies, including granular corneal dystrophy and Fuchs endothelial corneal dystrophy, are primarily caused by specific gene mutations that lead to abnormal deposition of structural proteins in different layers of the cornea or dysfunction of endothelial cells, resulting in corneal opacity or edema. Currently, phototherapeutic keratectomy (PTK) or corneal transplantation is commonly employed for clinical treatment of these conditions. However, despite these interventions, disease recurrence remains a possibility [140]. Therefore, gene therapy holds great promise in the treatment of corneal dystrophies. The fact that most corneal dystrophies exhibit autosomal dominant inheritance suggests that GS and gene editing strategies are the preferred approaches for these diseases. In contrast, inherited retinal degenerations (IRDs), including Leber congenital amaurosis (LCA) and RP, are primarily caused by mutations in one or more genes, leading to functional loss of photoreceptors or retinal pigment epithelial (RPE) cells, thereby impairing vision. For such loss-of-function ocular diseases, GRT is the most commonly employed gene therapy approach. In addition to the aforementioned rare inherited ocular disorders, gene therapy is increasingly being applied to common acquired eye diseases such as CNV, corneal fibrosis and retinal neovascularization. Innovative GS and gene replacement strategies offer the potential for etiological treatment of these conditions by addressing the underlying causes.

Hydrogel-mediated gene therapy represents an emerging and challenging area of research in the field of ophthalmology. However, this technology is still in the developmental stage; although active investigations are underway, published studies remain relatively limited. Summarizing the application of hydrogel-mediated gene therapy in ophthalmology is beneficial for promoting further advancement of this discipline. Given the unique anatomical features of the eye and the spatial specificity of therapeutic targets, this section will focus on current exploratory applications, outlining the status and potential of hydrogel-mediated gene therapy according to two major categories: ocular surface diseases and fundus diseases.

### Ocular surface diseases

The cornea, as the outermost transparent structure of the eye, plays a critical role in maintaining a normal intraocular microenvironment and a well-functioning visual system [141]. Corneal trauma is the most common form of corneal injury, and improper treatment may lead to the development of CNV. In severe cases, this can progress to ulceration or perforation, ultimately resulting in scarring and irreversible damage. CNV, as a common manifestation of ocular surface disease, is associated with a variety of conditions, including inflammation, infection, degenerative changes and trauma. Conventional eye drop therapies are limited by the corneal barrier, leading to poor bioavailability. As a result, the treatment of CNV continues to face challenges such as low drug delivery efficiency and poor patient compliance. In recent years, the cornea has emerged as an ideal target for gene therapy due to its superficial location and avascular, immune-privileged status [139]. Consequently, gene therapy has become an important strategy for promoting corneal repair, treating corneal fibrosis, managing CNV and combating corneal viral infections [142]. Hydrogel-mediated gene therapy strategies, by integrating the sustained-release properties of hydrogels with the precise targeting capabilities of gene therapy, offer an innovative therapeutic approach for ocular surface diseases and demonstrate excellent potential for clinical translation (Table 2).

**Table 2:** Application of hydrogel-mediated gene therapy in ocular surface diseases.

Diseases	Types of therapeutic genes	Classification of gene therapy technologies	Core materials	Ophthalmic disease applications	Reference
Ocular surface diseases	miR-150-5p	Gene silencing	GelMA	Promote the proliferation of corneal epithelial and stromal cells, inhibit the inflammatory response and ultimately realize multi-dimensional corneal repair	[143]
	miR-423-5p	Gene silencing	CHI (Chitosan)	Reduces extracellular matrix deposition, inhibits corneal scarring and promotes corneal epithelial regeneration and repair	[106]
	IGF-1	Gene replacing	HEMA and APMA	Overexpression of hIGF-I promotes cell proliferation and maintains corneal homeostasis	[144]
	miRNA 24-3p	Gene silencing	HA (hyaluronic acid)	Promotes corneal epithelial repair, inhibits inflammation and vascular proliferation	[145]
	TGF-β1 (siRNA)	Gene silencing	4-Arm-PEG-NHS	Promotes repair of corneal wounds and inhibits progression of corneal fibrosis	[146]

**Figure 4:**
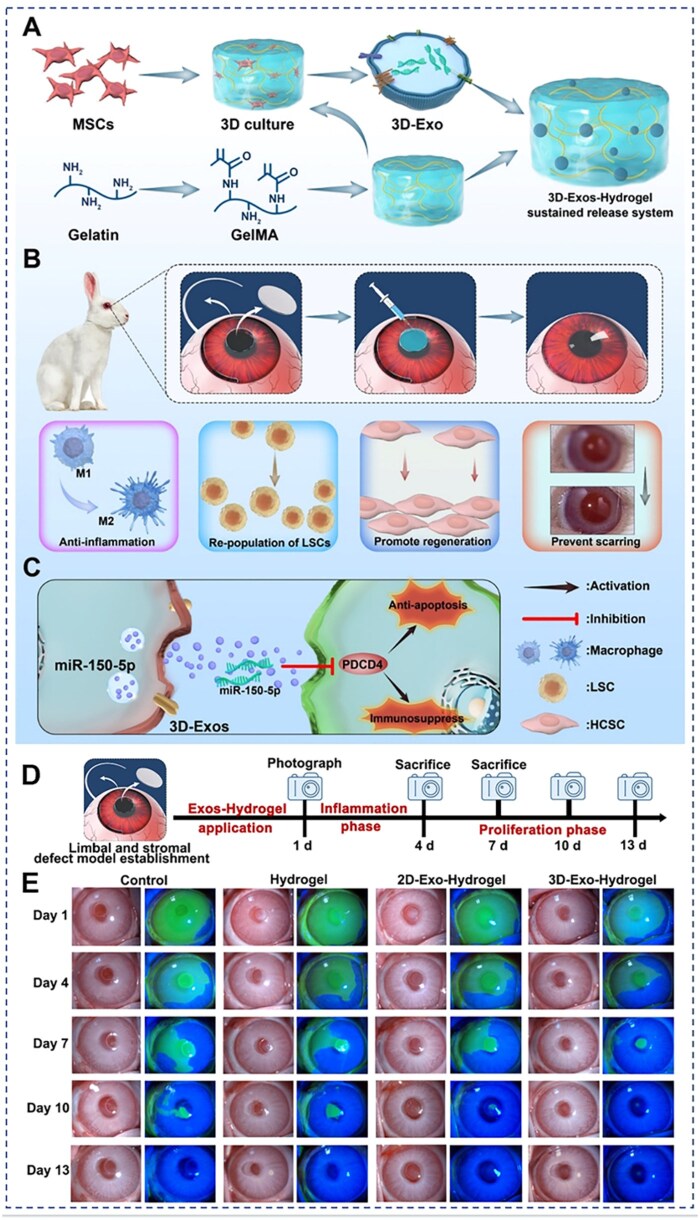
3D MSC exosome-functionalized hydrogels for corneal wound healing. (**A**) Preparation of the 3D-Exo-functionalized GelMA hydrogel. (**B**) Establishment of the rabbit model with corneal stromal defects and partial limbal stem cell deficiency (LSCD) and multiple functions of the 3D-Exo-hydrogel. (**C**) Schematic representation of how 3D-Exosomes promote corneal repair through the miR-150-5p/PDCD4 pathway. (**D**) Slit-lamp microscopy and fluorescein staining images of corneas following treatment with hydrogels containing 3D-Exosomes. (**E**) Slit-lamp microscopy and fluorescein staining images of corneas following treatment with hydrogels containing 3D-Exosomes. Reprinted with permission from Ref. [143]. Copyright 2025 Elsevier.

Exosome-based miRNA therapy is a common gene therapy approach that achieves post-transcriptional regulation of gene expression through targeted complementary pairing with mRNA, thereby inhibiting translation or inducing mRNA degradation [147–150]. Mesenchymal stem cell (MSC)-derived exosomes are known to maintain immune homeostasis and promote tissue repair and regeneration [151]. Based on this concept, Xu *et al.* successfully developed a methylacrylated gelatin (GelMA) hydrogel sustained-release system functionalized with three-dimensional exosomes (3D-Exos) enriched with miR-150-5p. This system targets PDCD4 to promote the proliferation of corneal epithelial and stromal cells, suppress inflammatory responses and ultimately achieve multidimensional corneal repair. Furthermore, the study demonstrated that, compared to conventional two-dimensional exosomes (2D-Exos), 3D-Exos are more effective in reducing inflammation and promoting corneal epithelial repair, thereby enhancing corneal formation and functional recovery. This improved performance may be attributed to the excellent biocompatibility of GelMA hydrogels and their porous three-dimensional network structure, which together facilitate large-scale expansion of MSCs and enhance their stemness. In this study, the hydrogel provided a three-dimensional scaffold for exosome growth via 3D culture and enabled prolonged sustained release of miR-150-5p through adhesion on the ocular surface, offering a novel gene therapy strategy for complex corneal diseases [143]. Overall, this study offers an innovative approach to corneal regenerative therapy and demonstrates the broad potential of hydrogel-mediated exosome delivery systems for gene therapy (Figure 4). However, the study has not yet conducted a systematic evaluation of the adhesive properties of the hydrogel on the ocular surface, which is critical for ensuring adequate retention of the therapeutic agents at the target site. Equally important is the precise control of the release kinetics of the hydrogel on the ocular surface, which remains a key scientific challenge to be addressed. In addition, future studies could further investigate the transfection efficiency and underlying mechanisms of miR-150-5p in corneal tissues, thereby promoting the development of gene therapy systems centered on miR-150-5p. Compared with exosomes, which are complex in origin, the use of purified miRNA loaded into controlled-release carriers holds promise for enhancing therapeutic targeting and stability. This strategy may also help to avoid the potential interference caused by nonessential RNAs, proteins and other components present in exosomes, thereby improving the precision and quality control of gene therapy for corneal diseases. Further exploration in these directions will lay a solid foundation for achieving safer, more effective and more controllable gene therapy for ocular surface diseases.

Tang *et al.* [106] developed a hydrogel sustained-release system (iPSC-MSC-Exos/CHI) by combining exosomes derived from iPSC-MSCs (iPSC-MSCs) with a thermosensitive chitosan (CHI) hydrogel. This system secretes miR-423-5p, which inhibits translocation-related membrane protein 2 (TRAM2) and downregulates the mRNA expression of collagen in the corneal stroma, thereby reducing extracellular matrix (ECM) deposition and preventing corneal scarring. In both a rat anterior segment injury model and a rat alkali burn model, the iPSC-MSC-Exos/CHI system not only promoted corneal epithelial regeneration and repair—similarly to the thermosensitive chitosan-based gel complex sustained-release system delivering stromal cell-derived factor 1-alpha (SDF-1α) but also demonstrated superior restorative effects on the corneal stroma. These findings highlight the powerful potential of gene therapy and provide a theoretical basis for thermosensitive hydrogel-mediated gene therapeutic strategies. In conclusion, this study ingeniously integrates the thermoresponsive and injectable properties of thermosensitive hydrogels by administering iPSC-MSC-Exos/CHI, thereby achieving localized sustained release of exosomes. Through the secretion of miR-423-5p, the system effectively promotes corneal repair, demonstrating great potential for clinical application in the treatment of corneal diseases. However, the current research is primarily based on *in vitro* experiments and animal models, and clinical trials in human corneal tissues have not yet been conducted. Therefore, the clinical feasibility and safety of this approach remain to be further validated. In addition, although the study identifies the miR-423-5p/TRAM2 signaling pathway as a key mechanism in corneal repair, the complex composition of exosomes suggests that additional molecular mechanisms may be involved. Future investigations combining multi-omics approaches could comprehensively identify the bioactive components within iPSC-MSC-Exos and elucidate their downstream regulatory pathways, thereby deepening our understanding of the underlying molecular mechanisms. Such efforts will facilitate the discovery of novel therapeutic targets and provide a theoretical foundation for the development of more precise and efficient gene therapy strategies for ocular surface diseases.

**Figure 5:**
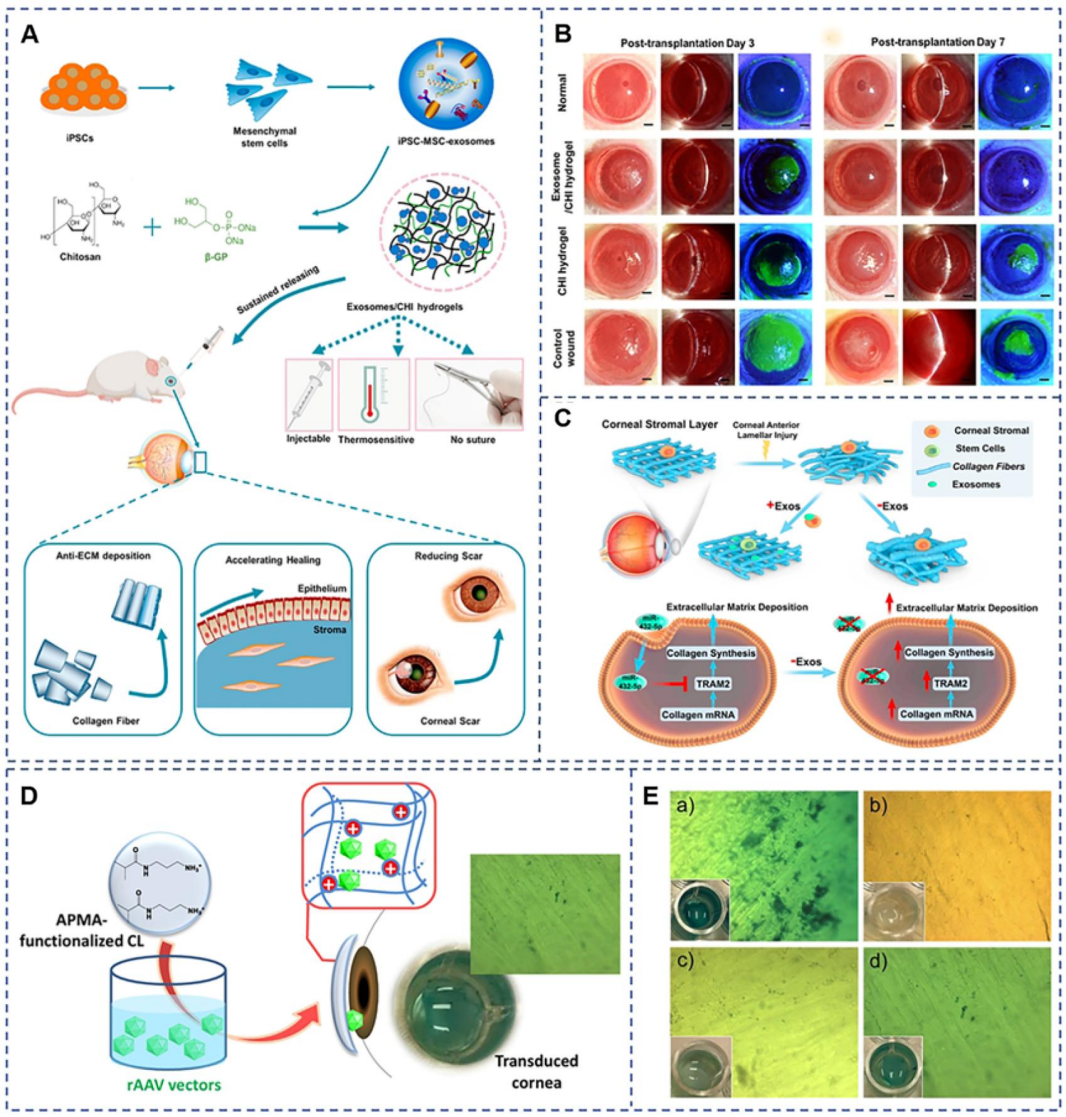
Exosomes-loaded thermosensitive hydrogels for corneal epithelium and stroma regeneration and controlled release of rAAV vectors from APMA-functionalized contact lenses for corneal gene therapy. (**A**) Preparation of the iPSC-MSC-exos hydrogels for corneal regeneration, along with its diverse therapeutic roles. (**B**) Slit-lamp imaging and fluorescein staining images of corneas following treatment with iPSC-MSC-Exos/CHI hydrogel. (**C**) The interaction between corneal stromal stem cells and exosomes during the ECM remodeling after corneal anterior lamellar injury. (A–C) Reprinted with permission from Ref. [106]. Copyright 2022 Elsevier. (**D**) Using hydrogel contact lenses as a host viral vector to achieve ocular transfection. (**E**) Transfected bovine corneas. (D, E) Reprinted with permission from Ref. [144]. Copyright 2020 MDPI.

Insulin-like growth factor-1 (IGF-1) is known for its role in promoting cell proliferation and maintaining corneal homeostasis. Recombinant adeno-associated virus (rAAV) vectors, owing to their small size and high transduction efficiency, have been widely applied in the treatment of various diseases [144, 152, 153]. However, the invasive administration methods associated with ocular gene delivery systems raise potential concerns, including the risks of intraocular inflammation and vitreous hemorrhage [154, 155]. Based on these issues, Fernando Alvarez-Rivera *et al.* designed a hydrogel contact lens composed of hydroxyethyl methacrylate (HEMA) and aminopropyl methacrylamide (APMA) for the delivery of the rAAV vector carrying hIGF-I (rAAV-hIGF-I). Experimental results demonstrated that this hydrogel contact lens possesses excellent transparency and oxygen permeability, ensuring a moist corneal surface. Additionally, the system enabled sustained rAAV release over 14 days without compromising gene transduction efficacy. Overexpression of hIGF-I via rAAV significantly enhanced cell proliferation. This innovative study developed a dual-purpose platform that not only serves as an rAAV delivery system for corneal gene therapy but also functions as a contact lens for the treatment of refractive errors, offering a promising noninvasive approach for ocular gene therapy [144] (Figure 5). However, this study still has certain limitations. Although it demonstrated that the hydrogel-based contact lenses possess good gene transduction capabilities, their therapeutic efficacy has not yet been evaluated in disease models using live animals. In addition, factors such as the comfort of lens wear, changes in tear film dynamics and the mechanical strength of the hydrogel have not been assessed. Future research should involve more comprehensive *in vivo* studies and integrate materials science approaches to further optimize the physicochemical properties of the hydrogel. Such efforts would enhance its biocompatibility, mechanical performance and controlled release capacity, thereby providing a solid theoretical and technical foundation for its clinical translation.

**Figure 6:**
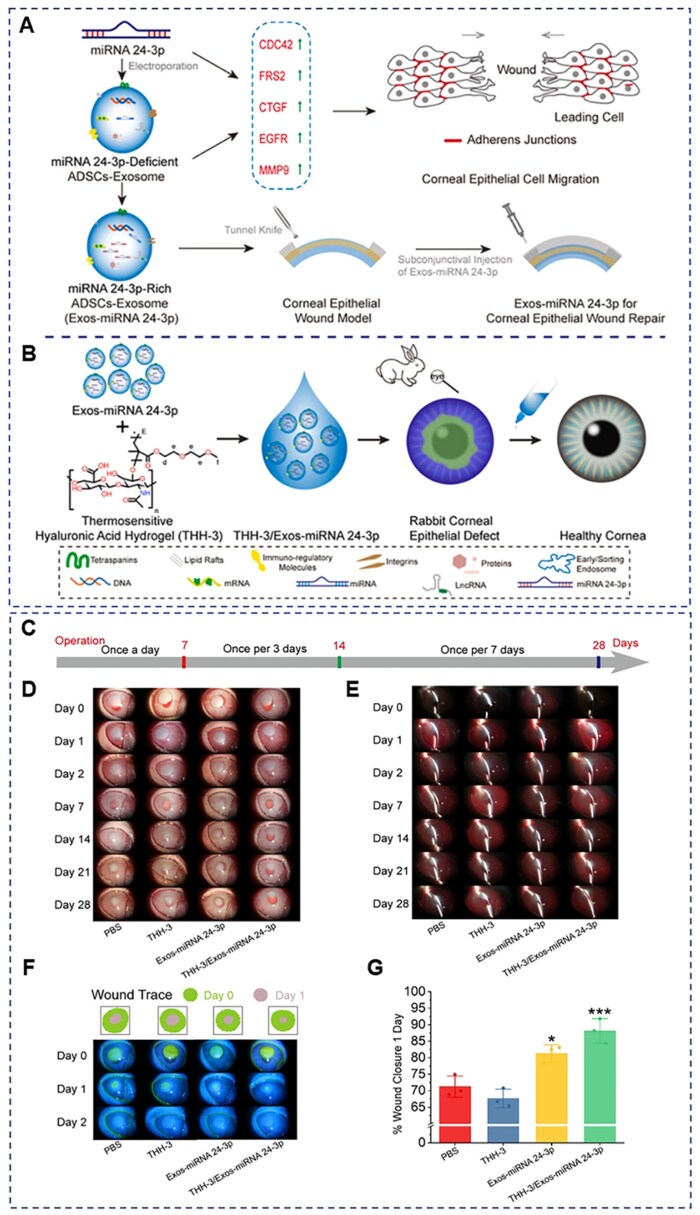
MiRNA 24-3p-rich exosomes functionalized DEGMA-modified hyaluronic acid hydrogels for corneal epithelial healing. (**A**) Preparation and application of miRNA 24-3p-rich exosomes. (**B**) Preparation and application of THH3/miRNA 24-3p-rich exosomes. (**C**) Prescription for eye medication. (**D** and **E**) Slit-lamp imaging, (**F**) wound trace and fluorescein staining images of corneas following treatment. (**G**) Wound closure rate 1 day after operation. Reprinted with permission from Ref. [145]. Copyright 2023 KeAi.

Hyaluronic acid is one of the natural components of the ECM, known for its excellent biocompatibility and having received U.S. FDA approval [156]. By modifying hyaluronic acid with di-(ethylene glycol) methyl ether methacrylate (DEGMA), its thermosensitive properties can be enhanced. Xiaomin Sun and colleagues developed a thermosensitive hyaluronic acid hydrogel (THH-3/Exos-miRNA 24-3p) enriched with miRNA 24-3p-loaded exosomes. Upon topical administration as eye drops, this system responds to the temperature of the cornea, forming an *in situ* gel that adheres closely to the corneal surface, thereby reducing the loss of bioavailability due to blinking and increasing corneal residence time. Moreover, the hydrogel promotes both *in vivo* and *in vitro* corneal epithelial repair while reducing inflammation and stromal fibrosis. Notably, *in vitro* co-culture experiments demonstrated that exosomes continued to be internalized by cultured corneal epithelial cells even on Day 7. In an alkali burn model, the THH-3/Exos-miRNA 24-3p sustained-release system exhibited superior efficacy in promoting corneal epithelial repair and suppressing inflammation and neovascularization compared to direct subconjunctival injection of exosomes [145]. The hyaluronic acid hydrogel enriched with engineered Exos-miRNA 24-3p, as a novel thermosensitive hydrogel carrier, exhibits excellent gelation capability on the ocular surface, allowing the formation of a stable gel structure locally. This facilitates the sustained release of exosomes and miRNA, effectively prolonging the retention time of active components on the ocular surface and significantly enhancing gene delivery efficiency (Figure 6). This hydrogel-mediated gene therapy, characterized by its noninvasive, precise and efficient nature, offers a novel therapeutic strategy for the treatment of corneal epithelial defects. In the future, further standardization of the hydrogel fabrication process and optimization of the gene release kinetics of the THH-3/Exos-miRNA 24-3p system will contribute to enhancing its controllability and stability, thereby accelerating its clinical translation in the field of ophthalmic gene therapy.

**Figure 7:**
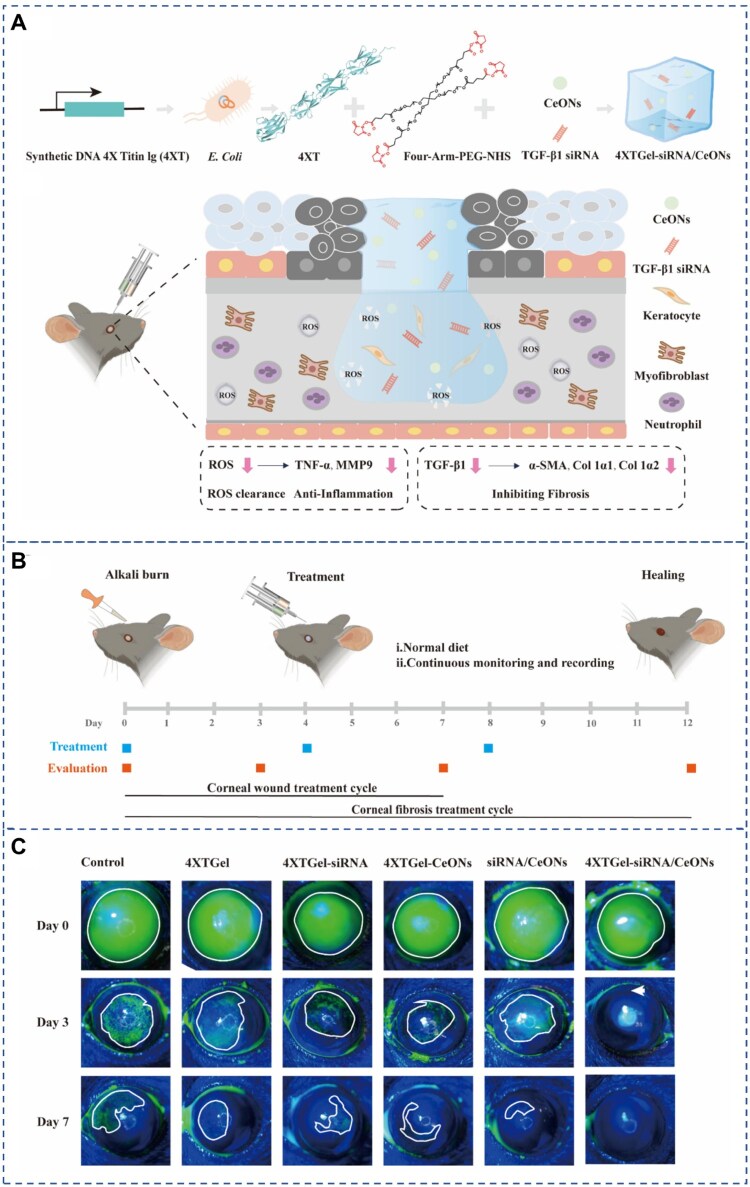
Composite synthetic protein hydrogel for inhibition of corneal fibrosis and treatment of corneal wounds. (**A**) The schematic diagram shows the preparation process and repair mechanism of 4XTGel-siRNA/CeONs. (**B**) Schematic diagram of the animal experiment time plan. (**C**) Representative images of corneal wound fluorescein staining. Reprinted with permission from Ref. [146]. Copyright 2025 Elsevier.

Severe complications such as corneal fibrosis may occur after ocular injury, leading to disordered stromal organization and reduced corneal transparency. Wang *et al.* developed a synthetic protein hydrogel based on recombinant proteins, into which cerium oxide nanoparticles (CeONs) with ROS scavenging activity and siRNA targeting the inhibition of transforming growth factor-β1 (TGF-β1) protein expression were incorporated. This hydrogel can undergo *in situ* gelation at the corneal defect site to form a stable scaffold. Through the synergistic effects of TGF-β1 suppression and ROS clearance, the system not only reduces oxidative stress and inflammation at the injury site and effectively promotes corneal wound healing in mice, but also inhibits the progression of corneal fibrosis [146]. This strategy, which combines therapeutically active inorganic nanozymes with gene therapy, enables synergistic blockade of corneal fibrosis through positive feedback mechanisms (Figure 7). Such a multimodal synergistic approach leverages the adhesive and supportive properties of photo-crosslinked hydrogels to achieve sustained release and combined effects of multiple therapeutic agents. As a result, therapeutic components that are otherwise rapidly cleared or prone to inactivation can be retained at the wound site for sufficient durations, thereby maximizing their therapeutic efficacy. This carrier design strategy—based on photo-crosslinked hydrogels embedded with nanovesicles and dual-target loading of functional components—offers a novel approach for addressing corneal fibrosis. It further highlights the promising clinical potential of hydrogel-mediated gene therapy. Future efforts focusing on optimizing the fabrication of nanoparticle-embedded hydrogels to enhance safety and biocompatibility, as well as standardizing photo-crosslinking parameters such as light intensity and exposure time, will facilitate the translation of this technology from the laboratory to clinical applications.

Advances in biotechnology have propelled gene therapy to the forefront of medicine [157], offering new hope for clinical treatment and steering it toward precision medicine [10]. Ocular surface diseases, due to their accessible location and the flexibility of treatment procedures, have attracted widespread attention from researchers [142, 158]. Although gene therapy for these conditions can be administered via eye drops, the limited conjunctival sac volume and ocular mechanical barriers result in low drug bioavailability [159, 160]. Due to its excellent biocompatibility, tunable physicochemical properties, adhesive capability and sustained release functionality, hydrogel has demonstrated unique potential in the field of ophthalmic gene therapy, particularly in the treatment of corneal epithelial defects, ocular surface inflammation and ocular surface neovascularization [161, 162]. Under pathological conditions such as corneal injury or post-surgical repair, the corneal barrier often undergoes varying degrees of structural or functional disruption, which creates favorable conditions for *in situ* hydrogel gelation and the localized action of gene carriers. By forming a gel directly at the lesion site, hydrogels take advantage of tissue exposure, thereby avoiding the need to forcibly penetrate an intact corneal barrier, while simultaneously prolonging the residence time of gene therapeutics on the ocular surface and improving their bioavailability. Current strategies for hydrogel-mediated gene therapy primarily focus on achieving effective encapsulation of gene drugs through specific functional modification of hydrogels, thereby improving their stability while increasing their residence time on the ocular surface, so as to exert long-term sustained-release and targeted therapeutic effects and enhance treatment efficiency [11]. However, several potential risks and challenges remain to be addressed. First, although the introduction of functional modification groups on the hydrogel surface can improve delivery performance, it may also trigger local immune responses, thus, compromising biocompatibility. Second, the intricate anatomical structure of ocular tissues imposes stringent requirements for the precise release of therapeutic genes. The hydrogel system is susceptible to interference from local microenvironmental factors, such as tear flow, blinking, and pH fluctuations, which can affect its controlled release behavior and targeting efficiency. Moreover, the adhesive strength, residence time and wearing comfort of hydrogels require further optimization. In addition, excessively large or heterogeneous pore sizes may result in gene entrapment or uneven release, thereby impacting therapeutic outcomes. These limitations highlight the need for future research to deepen the understanding of the pathogenesis and pathophysiological processes of ocular surface diseases, to guide the selection of appropriate gene therapy targets and the rational design of matching hydrogel carriers. Simultaneously, standardizing hydrogel fabrication processes is essential for improving the structural consistency and controlled release properties of hydrogel-based gene delivery systems. Through interdisciplinary integration of materials science, biomedicine and ophthalmology, the scope of hydrogel-mediated gene therapy for ocular surface disorders may be further expanded, ultimately facilitating its clinical translation.

### Fundus diseases

The eye is a complex yet highly refined organ, where damage to one tissue can affect others. The integrity of retinal structure and function is closely linked to optimal visual performance [163]. In recent years, with the rising incidence of posterior segment ocular diseases such as AMD, DR and RP, gene therapy has garnered increasing attention as a means to regulate pathological processes at their source. Compared with traditional viral vectors, hydrogels have emerged as a promising nonviral gene delivery strategy due to their excellent biocompatibility, tunable physicochemical properties, sustained release capabilities and injectability. In the context of retinal diseases, hydrogels can serve as carriers for gene therapy agents such as siRNA, enabling targeted treatment of retinal lesions through the modulation of their targeting properties and release kinetics (Table 3).

**Table 3:** Application of hydrogel-mediated gene therapy in fundus diseases.

Diseases	Types of therapeutic genes	Classification of gene therapy technologies	Core materials	Ophthalmic disease applications	Reference
Fundus diseases	siRNA	Gene silencing	LYTAC Plus	Achieving VEGFR degradation and ANG-2 silencing for neovascular n-AMD	[164]
	siMyD88	Gene silencing	CSGC	Synergistic utilization of siMyD88 anti-VEGF and Cu-PEI antioxidant properties to maintain the structure and function of the retina	[165]
	si-Cx43	Gene silencing	HAMA	Provides a synergistic and minimally invasive therapeutic strategy for DR by targeting angiogenesis, inflammation and neuroprotection with sustained drug delivery	[166]
	miR-21-3p	Gene silencing	HAMA	Provides both anti-angiogenic and neuroprotective effects to impede disease progression	[167]

**Figure 8:**
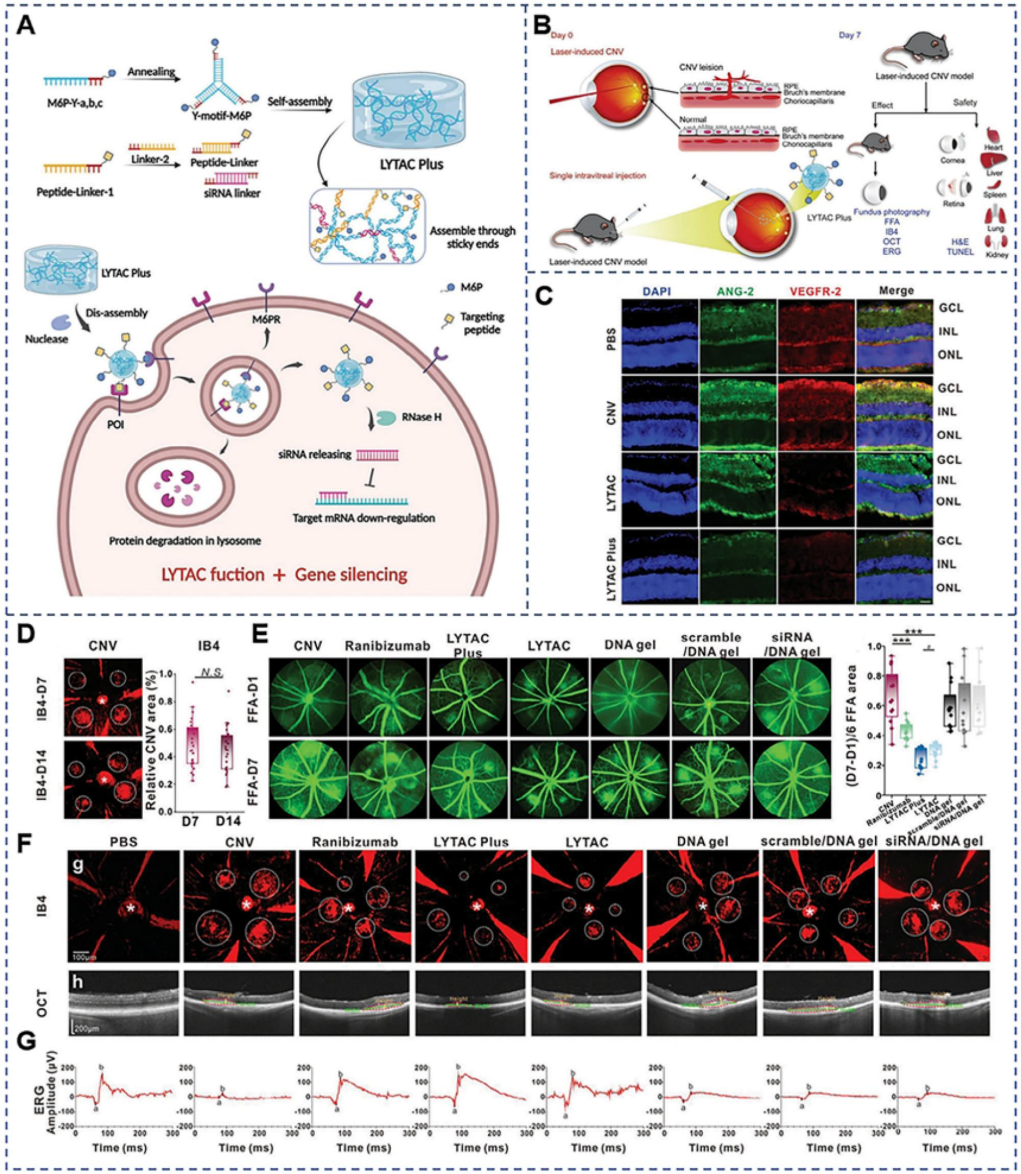
A nucleic acid-based LYTAC plus platform to simultaneously mediate disease-driven protein downregulation. (**A**) Preparation of the LYTAC plus hydrogel and its mechanisms of action. (**B**) Experimental design diagram of laser-induced CNV mouse model. (**C**) Immunofluorescence staining images of ANG-2 and VEGFR-2 in the retina of CNV mice. (**D**) Representative IB4 staining images and statistical analysis. (**E**) Representative FFA images and statistical analysis. (**F**) Representative IB4 staining and retinal OCT images. (**G**) Scotopic (dark-adapted) ERG responses. Reprinted with permission from Ref. [164]. Copyright 2024 Wiley-VCH.

In neovascular diseases, vascular endothelial growth factor A (VEGFA) plays a central role [168, 169]. Current treatment strategies for neovascularization primarily focus on anti-VEGFA therapies. Although traditional anti-VEGFA agents have shown some efficacy, they are limited by a short duration of action and instability [170, 171]. As a result, a multitude of gene therapy approaches targeting retinal neovascularization have emerged. Lysosome-targeting chimeras (LYTACs) are a cellular degradation technology similar to proteolysis-targeting chimeras (PROTACs), used for targeted protein degradation [140]. LYTACs primarily rely on the recognition of lysosome-targeting receptors (LTRs) to actively transport growth factors, disease-associated receptors and cytokines to lysosomes for degradation [172, 173]. However, unlike PROTACs, the degradation efficiency of LYTACs is generally lower. To address this limitation, Huang *et al.* integrated siRNA targeting angiopoietin-2 (ANG-2) into a LYTAC construct. By simultaneously targeting and degrading neovascularization-related genes such as VEGF/VEGFR and ANG-2, this approach enhances the therapeutic effect against n-AMD. In their study, Huang *et al.* introduced a peptide that binds to vascular endothelial growth factor receptors (VEGFR) and a mannose-6-phosphate (M6P) fragment into a self-assembled nucleic acid hydrogel to endow it with LYTAC functionality. Subsequent crosslinking with the aforementioned siRNA yielded the LYTAC Plus hydrogel. Experimental results demonstrated that following intravitreal injection, the LYTAC Plus hydrogel rapidly activated both LYTAC-based protein degradation and siRNA-mediated GS functions under the degradation of DNase I, resulting in the degradation of VEGFR and the silencing of ANG-2. Furthermore, a mouse model of n-AMD revealed that the LYTAC Plus hydrogel exhibited superior anti-angiogenic capabilities compared to the LYTAC hydrogel alone, underscoring its enhanced clinical potential [164]. Accordingly, the LYTAC Plus platform can be further customized to target other proteins and genes as needed, thereby promoting the advancement of precision and personalized medicine. In summary, the LYTAC Plus hydrogel achieves synergistic intervention of VEGFR-2 and ANG-2 by integrating protein degradation with GS, demonstrating significant therapeutic efficacy in the n-AMD model (Figure 8). However, this study did not evaluate the degradation rate of the hydrogel in the eye, the release kinetics of siRNA or long-term safety, which warrants further investigation into *in vivo* pharmacokinetics and long-term biosafety. Overall, this is a pioneering study that provides a novel strategy for hydrogel-mediated gene therapy targeting ocular fundus diseases, advancing the development of precision and personalized medicine. The LYTAC Plus hydrogel system also holds promise for extension to other retinal disorders in the future.

**Figure 9:**
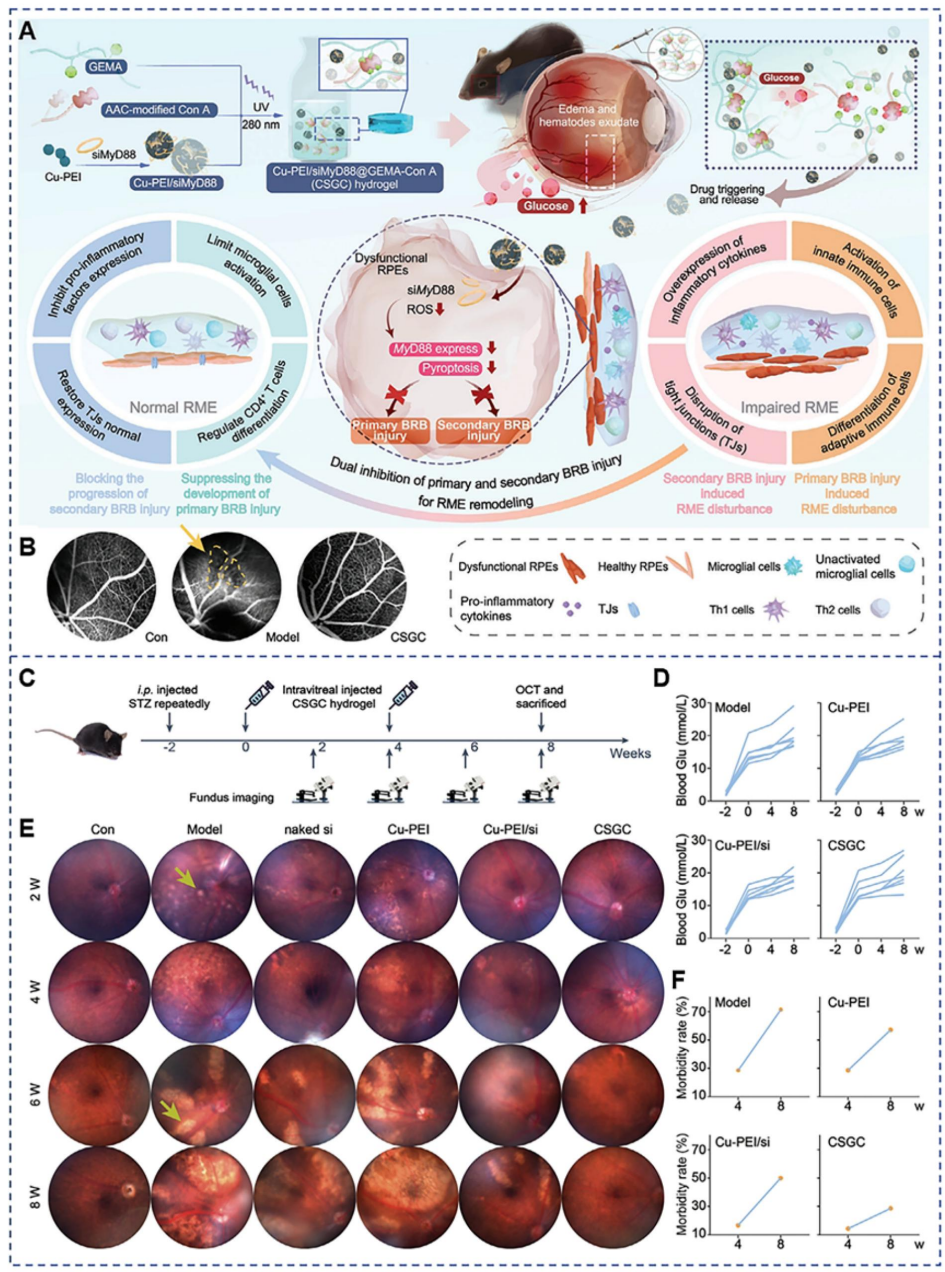
A glucose-responsive hydrogel inhibits primary and secondary BRB injury for retinal microenvironment remodeling in DR. (**A**) The schematic diagram shows the preparation process and mechanism of action of CSGC hydrogel. (**B**) Preliminary fluorescence detection of fundus angiography after CSGC hydrogel treatment. (**C**) Schematic diagram of the treatment plan for the experiment of CSGC hydrogel inhibiting the occurrence of DR In diabetic C57BL/6 mice. (**D**) Changes in blood glucose levels. (**E**) Representative retinal images after exposure to different treatments. (**F**) Respective morbidity rates of DR. Reprinted with permission from Ref. [165]. Copyright 2024 Wiley-VCH.

Diabetic retinopathy (DR), the most common blinding complication of diabetes, is pathologically characterized by hyperglycemia-induced retinal microvascular damage, pathological neovascularization and chronic inflammation. Glucose-responsive hydrogels represent a novel class of hydrogel systems capable of autonomously detecting changes in environmental glucose concentrations and undergoing corresponding structural transformations [174]. Zhou *et al.* synthesized a glucose-responsive hydrogel (CSGC) by co-polymerizing glucosyloxyethyl methacrylate (GEMA) with concanavalin A (Con A) loaded with Cu-polyethylenimine (Cu-PEI)/siMyD88. The key component of this hydrogel is Con A, which possesses multiple glucose-binding sites. By co-polymerizing GEMA with acrylic acid-modified Con A, a hydrogel network is established that disassembles in response to plasma glucose leaking from retinal vessels when the blood-retinal barrier (BRB) is compromised, thereby releasing Cu-PEI/siMyD88 nanoparticles (NPs). The hydrogel possesses both the potent antioxidant capacity of Cu‑PEI nanozyme and the ability to inhibit primary BRB injury. It can effectively scavenge ROS in the retina, suppress the expression of the pro-inflammatory cytokine IL-18 and attenuate the overactivation of microglia, thereby significantly reducing pyroptosis of RPE cells and preventing BRB disruption. Moreover, the CSGC hydrogel exerts sustained protective effects on the BRB by specifically silencing MyD88 expression in RPE cells and downregulating VEGF levels in the retina. Studies in diabetic mouse models demonstrated that the CSGC hydrogel can stabilize glucose levels in the retinal microenvironment, protect retinal cells from apoptosis, significantly reduce retinal edema and maintain both the structure and function of the retina [165]. In this study, the researchers synergistically harnessed siMyD88 for anti-VEGF effects and the antioxidant properties of Cu-PEI to develop an innovative glucose-responsive hydrogel gene therapy. By leveraging the hydrogel’s sustained-release properties, multimechanism synergy and targeted specificity, they significantly enhanced the efficacy of standalone gene therapy. This work lays the foundation for the future development of other combinatorial hydrogel-based gene therapy systems (Figure 9). Regrettably, this study did not provide precise data on the hydrogel’s degradation time under varying glucose concentrations and the release kinetics of the nanozyme and siRNA also require further quantitative validation. Future in-depth investigations into the degradation rate of the hydrogel and the release profiles of the nanozyme and siRNA will facilitate its potential application to other retinal diseases and promote advancements in the field of hydrogel-mediated gene therapy.

In addition, Shi *et al.* coupled si-Cx43–loaded nanoparticles (LPP-siRNA) with an anti-VEGF drug (Avastin) into a thermosensitive hydrogel to construct a si-Cx43-NPs@Avastin-hydrogel composite delivery system. This design innovatively co-loads si-Cx43 nanoparticles and Avastin into a thermosensitive hydrogel, enabling multidimensional combination therapy. First, si-Cx43 silences connexin 43 (Cx43), thereby exerting anti-inflammatory effects. Second, Avastin blocks VEGF-mediated angiogenesis. Third, the thermosensitive hydrogel acts as a drug reservoir that undergoes *in situ* gelation, protecting both therapeutics from enzymatic degradation and metabolic inactivation *in vivo*, while achieving sustained release of the two agents. This controlled release reduces dosing frequency and partially compensates for the limited efficacy of single-drug therapy. Overall, this composite system adopts a multi-target synergistic strategy of “anti-inflammation–anti-angiogenesis–barrier repair,” aiming to interrupt the mutually reinforcing cycle between inflammation and angiogenesis and to promote the restoration of retinal endothelial barrier function, thereby alleviating DR-related damage. This therapeutic approach overcomes the efficacy limitations of conventional anti-VEGF monotherapy and the drawbacks associated with repeated injections, providing a new paradigm for multi-target synergistic treatment of complex fundus diseases [166]. It should be noted that this study did not quantitatively evaluate the hydrogel degradation rate or drug loading capacity. Future work should further refine the systematic assessment of key parameters such as drug pharmacokinetics and material metabolism, which will be crucial for advancing this strategy from the laboratory to clinical application.

**Figure 10:**
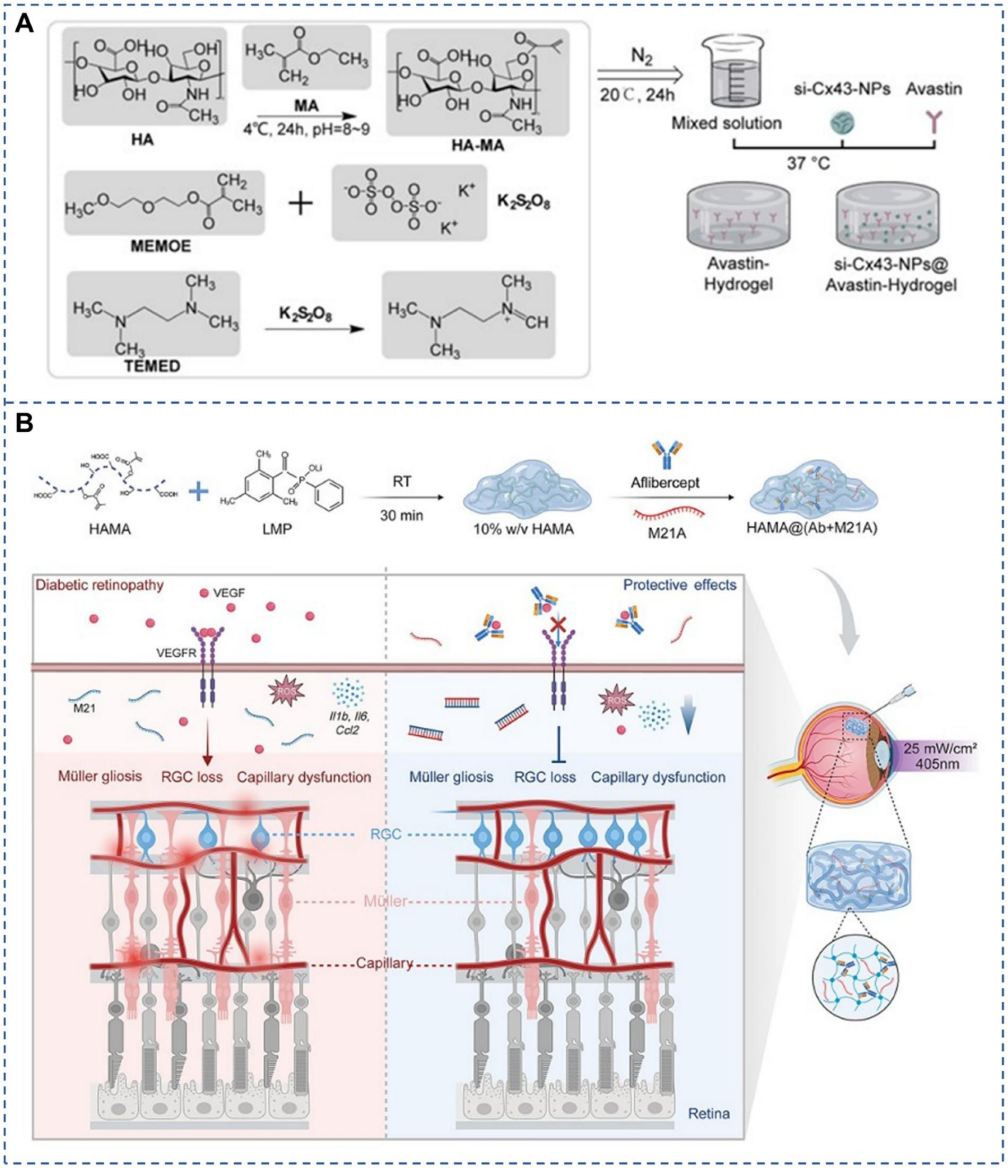
Hydrogel composite for synergistic treatment of DR. (**A**) Preparation and characterization of si-Cx43-NPs@Avastin-hydrogel. Reprinted with permission from Ref. [166]. Copyright 2025 Elsevier. (**B**) Schematic illustration of the preparation and dual therapeutic mechanisms of HAMA@(Ab+M21A). Reprinted with permission from Ref. [167]. Copyright 2025 Elsevier.

Considering that DR is accompanied not only by retinal vascular lesions but also by neuroinflammation and glial activation, conventional anti-VEGF therapy—while effective in controlling neovascularization—offers limited neuroprotection. Yin *et al.*, therefore, designed a photoresponsive HAMA hydrogel co-delivery system based on aflibercept and a miR-21-3p antagomir. This system aims to achieve combined intervention against retinal vascular dysfunction and neurodegeneration, thereby exerting synergistic therapeutic effects in DR. By simultaneously encapsulating anti-VEGF drugs and gene therapeutics within a photoresponsive hydrogel, this platform enables controllable regulation of drug release. Experimental results demonstrated that, following *in vivo* photo-crosslinking, the HAMA hydrogel exhibited sustained drug release for up to 45 days along with a controllable biodegradation profile. Both *in vitro* and *in vivo* studies further showed that HAMA@(Ab+M21A) improved vascular dysfunction, suppressed glial cell proliferation and promoted the survival of retinal ganglion cells [167] (Figure 10).

Due to the unique structure of the eye, treatment strategies for posterior segment diseases often rely on invasive methods such as intravitreal injections, which not only places high demands on operational accuracy, but also increases the risks and burdens associated with patients’ repeated medication administration [160, 175, 176]. Hydrogel-embedded nanoparticle delivery strategies offer a promising noninvasive approach for the treatment of posterior segment diseases, with the potential to partially overcome the limitations associated with invasive intravitreal therapies. Specifically, functionalization of hydrogels and their loaded nanocarriers with cell-penetrating peptides (CPPs) can markedly enhance barrier penetration efficiency [177]. In addition, hydrogel-embedded liposome eye-drop systems have emerged as an important noninvasive strategy for posterior ocular drug delivery. Hydrogels can form a stable adhesive layer on the ocular surface, prolonging drug residence time and enabling sustained release; meanwhile, chemical modification of liposomes, such as fluorination, further enhances their ability to traverse cellular membranes, thereby promoting drug penetration into intraocular tissues [178]. After reaching the intraocular environment, hydrogel-embedded nanoparticles can overcome the blood–retinal barrier (BRB) through multiple mechanisms. On the one hand, surface modification of nanoparticles with ligands that specifically bind to receptors on RPE cells enables receptor-mediated transcytosis, facilitating precise delivery of genes or drugs. On the other hand, nanoparticles with smaller sizes and optimized surface charge and hydrophilic–hydrophobic balance may cross the BRB via passive diffusion or transient modulation of tight junctions, allowing passage through intercellular spaces into the retina. Together, these mechanisms provide an important theoretical basis for hydrogel-mediated noninvasive delivery to the posterior segment of the eye.

Compared to conventional chemical therapies, gene therapy offers more sustained effects, thereby reducing the likelihood of repeated administrations to some extent [179, 180]. Owing to their excellent biocompatibility, degradable physical properties and modifiable chemical characteristics, hydrogels hold unique promise in the realm of gene therapy for posterior segment diseases. Although this approach has achieved certain progress, numerous challenges remain to be addressed. First, residual byproducts from the hydrogel synthesis process may pose a potential risk of inducing intraocular immune responses. Additionally, the clearance mechanisms of degradation products generated *in vivo* require further clarification, and the long-term safety of such materials on visual function remains insufficiently evaluated. Second, the retina is located in a deep and anatomically complex region, and is protected by the BRB. Although hydrogel-based delivery systems can reduce the frequency of intravitreal gene therapy injections, their efficiency in crossing the BRB and delivering genes to the posterior segment remains limited. Furthermore, some studies have attempted to achieve noninvasive gene delivery to the posterior segment of the eye by using hydrogel-embedded nanoparticles administered via the eye-drop route to penetrate the cornea; however, this route presents significant challenges in terms of dose control and therapeutic consistency, potentially increasing the risks associated with gene therapy. Therefore, future research should focus on reducing the immunogenicity of hydrogels, elucidating their metabolic pathways and assessing long-term safety, as well as combining nanotechnology engineering and molecular targeting strategies and developing hydrogel systems with enhanced targeting capabilities and controlled release profiles. Continued advancement in these areas may significantly expand the application boundaries of hydrogels in precision gene therapy for retinal diseases.

## Clinical translation challenges

Although hydrogel-mediated gene therapy has demonstrated broad application potential, it still faces significant challenges during the translational process from animal studies to clinical trials. Immune responses represent a critical consideration in gene therapy. As exogenous biomaterials, hydrogels may incorporate crosslinkers or functional groups during synthesis, which can be recognized by the immune system and potentially trigger inflammatory reactions or foreign body responses. Such immune activation may lead to accelerated clearance of the hydrogel carrier, thereby reducing gene delivery efficiency and the duration of therapeutic effects. In addition, immune-mediated reactions against hydrogel carriers may indirectly affect the associated gene cargo, potentially compromising gene stability or expression. Following degradation, residual monomers or degradation byproducts may further induce local inflammatory or immune responses during clearance, which could influence ocular tissue compatibility and treatment outcomes. Notably, the eye is considered an immune-privileged organ with distinct immunological characteristics. This unique environment may mitigate systemic immune responses and reduce the risk of widespread immune rejection of hydrogel gene complexes, making the eye a favorable site for gene therapy applications. However, localized immune reactions may still occur and warrant careful consideration.

In addition, immune-related processes may participate in the degradation of hydrogels, thereby influencing degradation rates, gene release profiles and overall therapeutic efficacy. Therefore, understanding and controlling the interactions between hydrogel materials, their degradation products, and immune responses are crucial for optimizing the clinical translation of hydrogel-mediated gene therapy. Long-term safety is another critical challenge. Gene therapy, by directly regulating pathogenic genes at the molecular level, holds the promise of achieving long-lasting therapeutic benefits with a single administration. Owing to their excellent sustained-release properties, hydrogels can enable continuous and stable delivery of gene therapeutics, thereby enhancing local bioavailability, reducing dosing frequency and to some extent improving treatment adherence and efficacy. However, these advantages are accompanied by potential risks. Incompletely degraded hydrogels or the long-term accumulation of degradation products *in vivo* may cause tissue toxicity and chronic inflammation, potentially triggering immune rejection responses. Moreover, if sustained gene expression is not precisely regulated, uncontrolled overexpression or insufficient expression may lead to adverse effects, induce unintended biological responses and even pose a risk of genetic mutations.

Conversely, the transition of hydrogel-mediated gene therapy from laboratory research to large-scale clinical trials also faces challenges related to scalable manufacturing. Ensuring the quality and reproducibility of hydrogels, as well as achieving standardized and scalable production processes, are essential considerations for advancing hydrogel-based gene therapy strategies toward clinical application.

In summary, although hydrogel-mediated gene therapy holds substantial promise, several challenges remain before its successful clinical translation, including immune responses, long-term safety concerns and large-scale manufacturing issues. Addressing these challenges will require multidisciplinary collaboration and in-depth research across biomaterials science, genetic engineering and manufacturing technologies. Breakthroughs in immune evasion, carrier optimization and process standardization will be critical for driving hydrogel-mediated gene therapy toward clinical implementation.

## Conclusion and prospect

Hydrogel-mediated gene therapy presents unique advantages and holds significant promise for the treatment of ophthalmic diseases. In recent years, researchers have harnessed the controlled-release properties, targeted local delivery capabilities and excellent biocompatibility of hydrogels to achieve efficient delivery and sustained expression of therapeutic nucleic acids—such as siRNA and miRNA—to ocular target tissues including the cornea and retina. This strategy effectively reduces dosing frequency and enhances therapeutic efficacy, thereby driving meaningful progress in the field of ocular gene therapy and laying a solid foundation for the clinical translation of hydrogel-mediated gene therapy systems.

In addition, as gene therapy for ophthalmic diseases continues to advance, the introduction of personalized medicine concepts offers greater developmental potential for this field. Personalized medicine emphasizes tailoring therapeutic strategies based on each patient’s unique genetic background, immune system characteristics and disease progression. In the context of hydrogel-mediated gene therapy, personalized medicine can further assist in optimizing treatment strategies, enhancing therapeutic efficacy and reducing the risk of adverse side effects.

Currently, hydrogel-mediated gene therapy has made some progress in ophthalmology, predominantly in acquired diseases such as corneal injuries and DR. In contrast, ocular genetic disorders such as LCA and RP have been extensively studied using viral vectors, while research on hydrogel-based nonviral vectors remains limited. This disparity may be attributed to the highly mature nature of viral vectors like AAV in ocular gene therapy, whereas hydrogels, as emerging nonviral carriers, continue to face several challenges that constrain their broader application. These include low gene loading efficiency, difficulties in delivering nucleic acids to the cell nucleus leading to low transfection efficiency, challenges in achieving precise single-gene targeting and potential immune risks associated with long-term hydrogel retention. Addressing these challenges is essential for advancing hydrogel-based gene therapy platforms toward broader clinical applications and for realizing their full potential in treating a wider range of ophthalmic disorders.

These challenges also highlight the importance of personalized medicine. Personalized therapeutic strategies can optimize hydrogel design and integrate more precise gene-editing tools based on individual patient characteristics and clinical needs, thereby improving hydrogel-mediated gene delivery systems to achieve optimal therapeutic outcomes. In addition, personalized medical approaches enable immune monitoring of patients receiving hydrogel-mediated gene therapy, allowing immunomodulatory strategies to be adjusted accordingly to ensure the long-term stability and effectiveness of gene therapy.

With an increasingly deep understanding of gene function and regulatory mechanisms, breakthroughs in hydrogel-mediated gene therapy for ophthalmic diseases can be anticipated through several avenues by optimizing material types, improving crosslinking methods and expanding gene modification techniques: (1) Development of smart hydrogels. Create intelligent hydrogels that respond to the intraocular microenvironment, enabling on-demand and precise gene release at the target site. This approach aims to enhance the efficiency of gene loading and utilization. (2) Design of novel hydrogel materials. Engineer new hydrogel formulations that not only protect genes or gene editing tools but also improve their delivery efficiency, thereby overcoming current limitations in nucleic acid stability and cellular uptake. (3) Combination systems for sustained release and immunosuppression. Explore integrated systems that couple hydrogel-based sustained release with immunosuppressive strategies. This dual approach could extend the therapeutic duration of the gene therapy while mitigating intraocular immune rejection responses. (4) Multimodal synergistic therapies. Investigate the integration of gene therapy with anti-angiogenic agents, anti-inflammatory and antioxidant factors. Such combinatorial strategies aim to enhance treatment efficacy across multiple dimensions, addressing the complex pathologies of ocular diseases. (5) Synergy between hydrogels and other delivery platforms. Leverage the complementary strengths of hydrogels, nanoparticles or viral vectors. Through the personalized selection of smart hydrogel types and the integration of combinatorial therapeutic strategies, hydrogel-mediated gene therapy can be flexibly adjusted according to a patient’s specific disease condition and intraocular environment, thereby achieving more efficient therapeutic outcomes. These strategies offer promising directions for overcoming current challenges and expanding the clinical applicability of hydrogel-mediated gene therapy in the treatment of various ophthalmic conditions.

In summary, hydrogels serve as an innovative platform for ocular gene therapy, with their flexible functional design significantly advancing the field. With the continued convergence and interdisciplinary progress of materials science, gene engineering and biomedicine, this area holds great promise for delivering safer, more efficient and precision-targeted treatments for a broader spectrum of ocular diseases.

## Data Availability

Data will be made available on request.
